# Harnessing natural killer cells for refractory/relapsed non-Hodgkin lymphoma: biological roles, clinical trials, and future prospective

**DOI:** 10.1186/s40364-024-00610-z

**Published:** 2024-07-17

**Authors:** Mehdi Bakhtiyaridovvombaygi, Somayeh Yazdanparast, Setare Kheyrandish, Seyed Mehrab Safdari, Fateme Amiri Samani, Mahsa Sohani, Akram Sadat Jaafarian, Fateme Damirchiloo, Amirhossein Izadpanah, Sahar Parkhideh, Fatemeh Mikanik, Elham Roshandel, Abbas Hajifathali, Ahmad Gharehbaghian

**Affiliations:** 1 Student Research Committee, Department of Hematology and Blood Banking, School of Allied Medical Sciences, Shahid Beheshti University of Medical Sciences, Tehran, Iran; 2 Hematopoietic Stem Cell Research Center, Shahid Beheshti University of Medical Sciences, Tehran, Iran; 3 Department of Hematology and Blood Banking, Faculty of Medical Sciences, Tarbiat Modares University, Tehran, Iran; 4 Departments of Hematology and Blood Transfusion, School of Allied Medicine, Iran University of Medical Sciences, Tehran, Iran; 5 Blood Transfusion Research Center, High Institute for Research and Education in Transfusion Medicine, Iranian Blood Transfusion Organization (IBTO), Tehran, Iran; 6 Laboratory Hematology and Blood Bank Department, School of Allied Medical Sciences, Shahid Beheshti University of Medical Sciences, Tehran, Iran; 7 Pediatric Congenital Hematologic Disorders Research Center, School of Medicine, Shahid Beheshti University of Medical Sciences, Tehran, Iran

**Keywords:** Non-Hodgkin lymphoma, Natural killer cells, Immunotherapy, Chimeric antigen receptors, Monoclonal antibodies

## Abstract

Non-Hodgkin lymphomas (NHLs) are heterogeneous and are among the most common hematological malignancies worldwide. Despite the advances in the treatment of patients with NHLs, relapse or resistance to treatment is anticipated in several patients. Therefore, novel therapeutic approaches are needed. Recently, natural killer (NK) cell-based immunotherapy alone or in combination with monoclonal antibodies, chimeric antigen receptors, or bispecific killer engagers have been applied in many investigations for NHL treatment. The functional defects of NK cells and the ability of cancerous cells to escape NK cell-mediated cytotoxicity within the tumor microenvironment of NHLs, as well as the beneficial results from previous studies in the context of NK cell-based immunotherapy in NHLs, direct our attention to this therapeutic strategy. This review aims to summarize clinical studies focusing on the applications of NK cells in the immunotherapy of patients with NHL.

## Introduction

B-cell non-Hodgkin lymphoma (NHL) collectively represents the most common type of hematologic malignancy [1]. While advances in chemotherapy, monoclonal antibodies, and stem cell transplantation have improved survival rates, many NHL patients remain resistant to therapy or experience relapse. This highlights the necessity for finding novel curative therapeutic options for these patients [2–4].

Recently, novel therapeutic approaches, such as chimeric antigen receptor (CAR)-T cell therapy, have been utilized in several clinical trials for patients with relapsed/refractory (R/R) B-cell NHL, resulting in promising clinical responses [5–7]. However, this therapeutic approach is expensive and associated with unique and severe side effects such as cytokine release syndrome (CRS), immune effector cell-associated neurotoxicity syndrome (ICANS), and graft-versus-host disease (GVHD) in allogenic settings [8, 9]. Other immune cells, such as natural killer (NK) cells, which exhibit significant cytotoxic activity against cancer cells and possess a safer immune profile, can be used as alternative approaches for immunotherapy of patients with R/R NHL. Moreover, it is also feasible to equip NK cells with a CAR structure [10, 11].

This study will delve into the B-cell NHL tumor microenvironment (TME) and the interaction between NK cells and malignant cells. Additionally, we provide a comprehensive review of clinical trials focused on the utilization of NK cells in patients with R/R NHL. Finally, new approaches recently used to increase NK cells effectiveness for B-cell NHL immunotherapy are summarized.

## NK cells: biology, receptors, and functions

NK cells, a specialized subset of innate lymphoid cells (ILCs), can distinguish between self-cells and non-self-cells through the recognition of self-major histocompatibility complex (MHC) I molecules [12, 13]. They constitute approximately 10–15% of the lymphocyte population in peripheral blood, and characterized as large granular lymphocytes with kidney-shaped nuclei, a high cytoplasm-to-nucleus ratio, and large azurophilic granules in cytoplasm [14, 15]. NK cell development and maturation primarily occur in the bone marrow, where common lymphoid progenitors (CLPs) differentiate into NK precursors (NKPs), immature NK cells, and finally mature NK cells [12]. Notably, recombinant interleukin (rIL)-15 plays a crucial role in NK cell development from hematopoietic stem cells [16]. In humans, CD122 expression on NKPs is crucial for NK cell lineage commitment, and CD56 expression is a final step in the differentiation of NKPs into NK cells [17]. NK cells are typically identified by the expression of CD56 and CD16, and the absence of CD3 (T cell marker) [18].

Human NK cells can be classified into two main subsets: the CD56^bright^ subset, which is characterized by immaturity, limited cytolytic activity, but high cytokine production; and the CD56^dim^ subset, which is mature, exhibits higher cytolytic activity, but lower cytokine production [19].

NK cells are equipped with various germline-encoded activating and inhibiting receptors [20]. The function of NK cells is delicately regulated by the balance of the activating and inhibitory signals that are transmitted through their receptors [21]. Table 1 provides an overview of NK cell receptors.

**Table 1:** Overview of NK cells receptors

Receptors	Molecular structure	CD marker	Ligand (s)
**Activating receptors**			
**HLA-specific activating receptors**	Immunoglobin superfamily		
(I) Killer immunoglobin receptors (KIRs)			
• KIR2DS1		CD158h	HLA-C2
• KIR2DS2		CD158j	HLA-C1
• KIR2DL4		CD158d	HLA-G
• KIR2DS5		CD158g	?
• KIR3DS1		CD158e2	HLA-F
(II) CD94/NKG2	C-type lectin family		
NKG2C		CD159c	HLA-E
NKG2E		CD159e	?
**Non-HLA-specific activating receptors**			
(I) Natural cytotoxicity receptors (NCRs)	Immunoglobin superfamily		
• NKp46 (NCR1)		CD335	Heparin, viral HA and HN
• NKp44 (NCR2)		CD336	viral HA and HN, NKp44L, PCNA
• NKp30 (NCR3)		CD337	B7-H6, BAT3, viral HA
(II) NKG2D	C-type lectin	CD314	MIC-A, MIC-B, ULBP
(III) Coreceptors			
• 2B4	Immunoglobin superfamily	CD244	SLAMF2 (CD48)
• NTB-A	Immunoglobin superfamily	CD352	NTB-A
• DNAM-1	Immunoglobin superfamily	CD226	PVR (CD155), Nectin-2 (CD112)
• NKp80	C- type lectin-like family	-	AICL
(IV) FcγRIII	Immunoglobin superfamily	CD16	IgG1, IgG2, IgG3
**Inhibitory receptors**			
**HLA-specific inhibitory receptors**			
(I) Killer immunoglobin receptors (KIRs)	Immunoglobin superfamily		
• KIR2DL1		CD158a	HLA-C2
• KIR2DL2		CD158b	HLA-C1
• KIR2DL3		CD158b2	HLA-C1
• KIR2DL5		CD158F	**?**
• KIR3DL1		CD158e	HLA-Bw4
• KIR3DL2		CD158k	HLA-A3, A11
(II) CD94/NKG2	C-type lectin family		
• NKG2A		CD158a	HLA-E
(III) Other			
• LIR-1(ILT2)	Immunoglobin superfamily	CD85	?
**Non-HLA-specific inhibitory receptors**			
(I) PD-1	Immunoglobin superfamily	CD279	PD-L1/PD-L2
(II) Siglec7/p75/AIRM1/	Immunoglobin superfamily	CD328	Sialic acid
(III) LAIR-1/p40	Immunoglobin superfamily	CD305	Collagen, C1q, SP-D, ADP
(IV) IRp60	Immunoglobin superfamily	CD300a	?

*HN* Hemagglutinin neuraminidases, *PVR* Poliovirus receptor, *AICL* Activation-induced C-type, *SP-D* Surfactant protein D, *ADP* Adiponectin, *PCNA* Proliferating cell nuclear antigen, *SLAMF2* Signaling lymphocytic activation molecule 2, *MIC* MHC class I chain-related protein, *ULBP* UL16 binding protein 1

NK cells express the HLA-specific activating receptors such as KIRs/CD158 (2DS1–2DS5 and 3DS1), NKG2C, and NKG2E. NKG2C and NKG2E are expressed as heterodimers with CD94. Upon interaction with HLA-E, they transmit activating signals through the DNAX-activation protein (DAP)-12 adaptor molecule [22–24]. The natural cytotoxicity receptors (NCRs) including NKp46, NKp44, and NKp30 are the major non-HLA specific activating NK receptors, which evoke an immune response upon detection of cognate viral and cellular ligands [20, 25]. NKG2D is another non-HLA-specific activating NK cell receptor [26]. The UL16-binding protein (ULBP) and MHC class I chain-related proteins A and B (MICA/B), which are increased in the tumor, stressed, and infected cells, are representative of NKG2D ligands [27]. Additionally, some other molecules, such as 2B4, NTB-A, CD59, NKp80, and DNAX accessory molecule-1 (DNAM-1), are essentially coreceptors; in fact, they can intensify the NK cell triggering induced by NCRs or NKG2D (See Table 1) [28–32]. NK cells are also equipped with the CD16a (FcγRIIIa, a low-affinity Fc), which plays a crucial role in their antibody-dependent cell-mediated cytotoxicity (ADCC) effector function. It is worth noting that CD16 is the only receptor that can activate NK cells without the need for further activation from other receptors [33].

Besides activating receptors, NK cells express inhibitory receptors that modulate the strength of activating receptors and contribute to regulating immune responses and tolerance [34]. The CD94/NKG2A (CD94/CD159a) heterodimer and members of the KIR/CD158 family are two distinct classes of HLA-specific inhibitory receptors [35, 36]. The LIR-1/ILT2/CD85 is an inhibitory receptor with broad specificity for both classical and non-classical MHC molecules [37–39]. Program-cell death receptor 1 (PD-1), Sialic acid recognizing Immunoglobulin-like Lectins (Siglecs)/p75/AIRM1/CD328, leukocyte-associated immunoglobulin-like receptor-1 (LAIR-1)/p40/CD305, and IRp60/CD300a known as another non-HLA-specific inhibitory receptor that hamper NK cell-mediated antitumor immunity through the recognition of different ligands on the surface of cancerous cells (See Table 1) [40, 41].

**Fig. 1:**
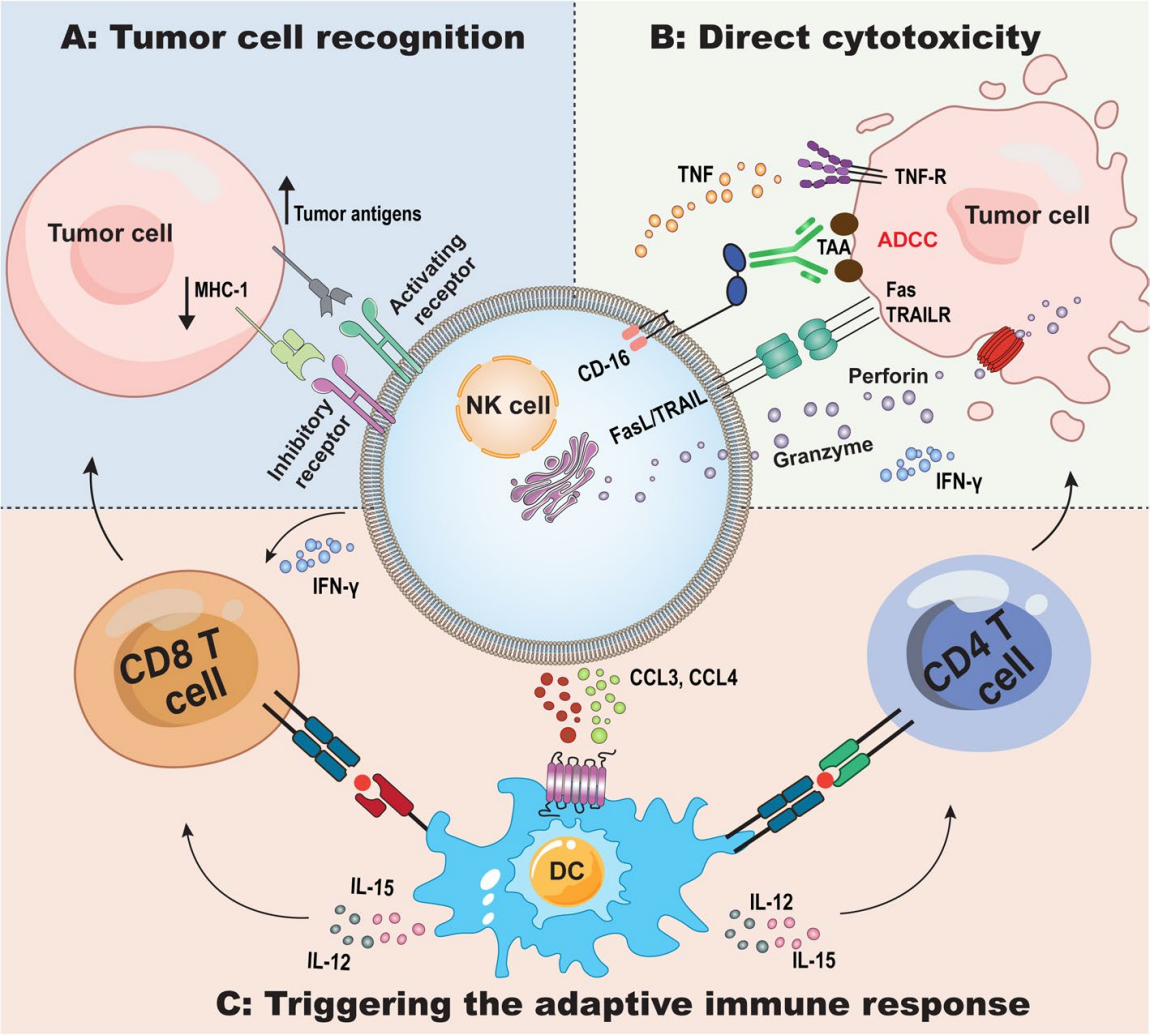
Natural killer (NK) cell function within tumor microenvironment (TME). A) Missing-self recognition against tumor cells lacking MHC class I ligands for inhibitory NK receptors. B) Direct cytotoxicity against tumor cells mediated by releasing cytotoxic granules containing perforin/granzymes, IFN-γ and TNF-α production, antibody-dependent cell-mediated cytotoxicity (ADCC) via CD16, and induction of apoptosis pathway through death receptor ligands like TRAIL/FasL. C) Triggering the adaptive antitumor immunity by recruiting dendritic cells via chemokines and then amplifying CD4^+^ and CD8^+^ T cells antitumor immune response

NK cells have diverse functions, including their natural antitumor and antiviral activities, as well as their regulatory roles in modulating immune responses and promoting tissue growth. They are abundant in the TME, where they kill cancer cells in a variety of ways [21]. The antitumor functions of NK cells include missing self-mechanisms, direct cytotoxicity, and activation of adaptive immune responses (Fig. 1) [42–47].

## TME in NHLs: composition and functions

**Fig. 2:**
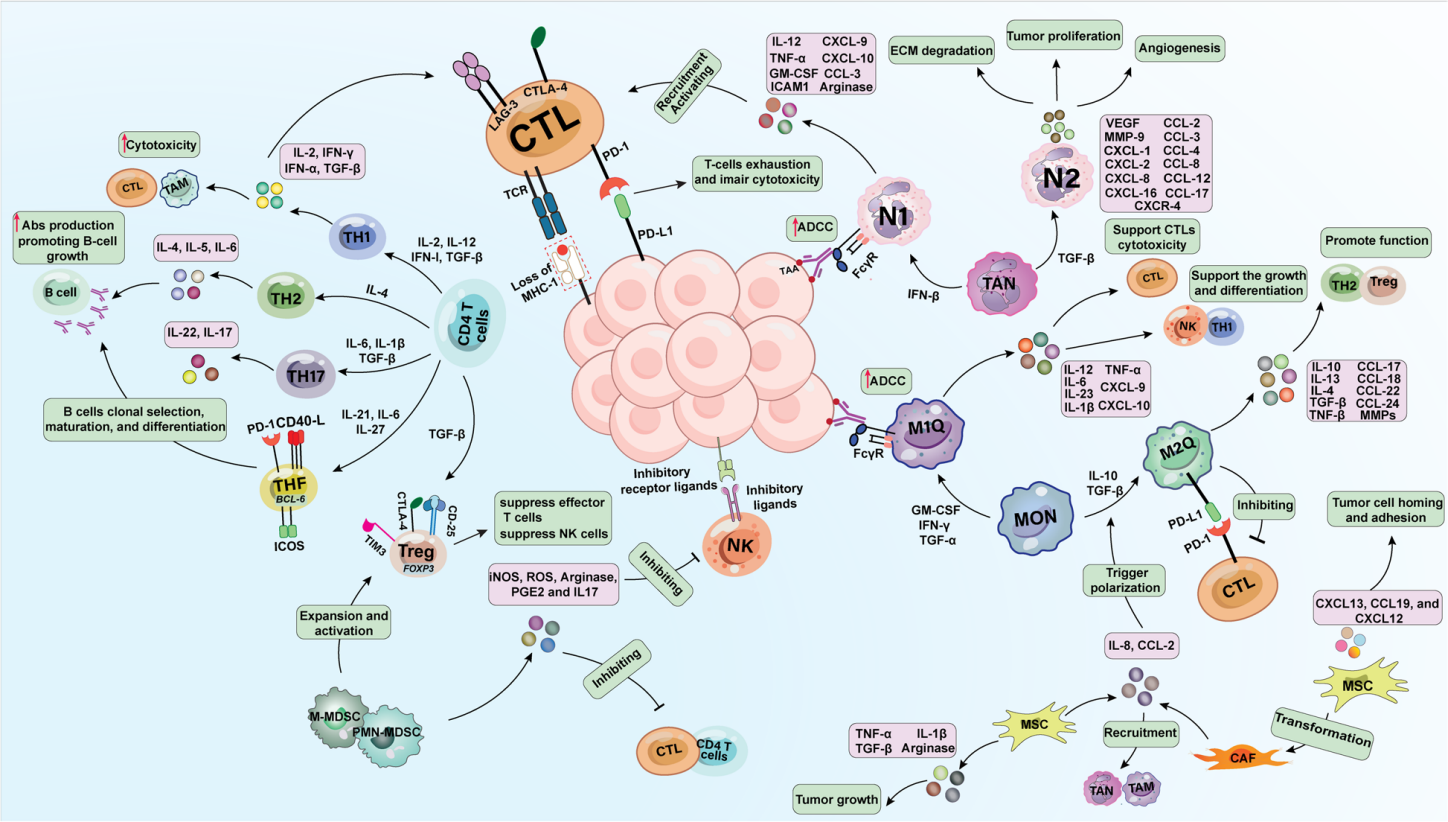
Schematic representation of tumor microenvironment (TME) constituents in non-Hodgkin lymphomas (NHLs). The TME comprises cellular and noncellular components. The cellular microenvironment consists of immune and nonimmune cells that can play pro- or antitumorigenic roles within the NHL milieu (see Table 2 for more details about the pro-tumorigenic and anti-tumorigenic cells in the TME). CTLs, effector CD4.^+^ T cells (TH1, TH2, TH17, TFH, and Treg cells), follicular dendritic cells (FDCs), natural killer (NK) cells, tumor-associated macrophages (TAMs), tumor-associated neutrophils (TANs), and myeloid-derived suppressor cells (MDSCs) constitute the immune microenvironment. On the other hand, mesenchymal stromal cells (MSCs) and cancer-associated fibroblasts (CAFs) are involved in nonimmune microenvironments. The noncellular components include the extracellular matrix (ECM) as well as various cytokines, chemokines, and molecules produced by cancerous and noncancerous cells and executed by these cells to induce their stimulatory or inhibitory effect on bystander cells (see Sect. "TME in NHLs: Composition and Functions" for more details about the interaction between cells inside the TME)

The development of B-cell lymphoma involves an intricate interplay between tumor cells and the surrounding TME (Fig. 2). The microenvironment in B-cell lymphoma is fascinating because it has crucial functions in regulating the survival and growth of tumor cells, promoting immune evasion, and contributing to the development of resistance to treatment [48–52]. It is worth mentioning that different cells within the TME can display pro-tumorigenic or anti-tumorigenic functions, as shown in Table 2.

**Table 2:** Overview of pro-tumorigenic and anti-tumorigenic cells in tumor microenvironment (TME)

**Anti-tumorigenic cells**			
**TH1 cell**	• **Markers:** CD4^+^, TCR^+^, CXCR3^+^, CCR5^+^	**TH17 cell**	• **Markers:** CD4^+^, TCR^+^, CCR6^+^
	• **Polarization cytokines:** IL-12, IFN-I		• **Polarization cytokines:** IL-6, IL-1β, TGF-β
	• **Mechanisms:** Produce cytokines (IFN-α, TGF-β, IL-2, INF-γ), Increase cells’ function (CTLs and macrophages)		• **Mechanisms:** Produce cytokines (IL-17, IL-22), Increase inflammation
**M1 macrophage**	• **Markers:** CD68^+^, CD80^+^, CD86^+^	**N1 neutrophil**	• **Markers:** CD16^+^, CD66b^+^, CD170^low^
	• **Polarization cytokines:** IFN-γ, TGF-α, GM-CSF		• **Polarization cytokines:** INF-β
	• **Mechanisms:** Produce cytokines (IL12, IL-6, IL-1β, IL-23, TNF-α), Produce chemokines (CXCL9, CXCL10), Increase cells’ function (TH1 cells, CTLs, and NK cells), Increase inflammation		• **Mechanisms**: Produce cytokines (IL-12, TNF-a, GM-CSF), Produce chemokines (CXCL9, CXCL10, CCL-3), Produce ICAM-1, produce arginase, Increase CTL activation and recruitment, Mediate ADCC
**NK cell**	• **Markers:** Immature NK cells: CD56^bright^, CD16^−^ Mature NK cells: CD56^dim^, CD16 + • **Polarization cytokines: -**	**CTL**	• **Markers:** CD8^+^, TCR^+^
			• **Polarization cytokines: -**
	• **Mechanisms:** Produce cytokines (IFN-γ, TNF-α, GM-CSF, IL-10, IL-5, and IL-13), Produce chemokines (MIP-1α, MIP-1β, IL-8, RANTES), Produce perforin and granzymes, Mediate ADCC, Mediate missing-self mechanisms		• **Mechanisms:** Produce cytokines (IFN-γ, TNF-α), Produce death-induces granules (granzymes, perforin, cathepsin C, and granulysin), Express death ligand (FASL)
**Pro-tumorigenic cells**			
**TH2 cell**	• **Markers:** CD4^+^, TCR^+^, CCR34^+^, CCR8^+^	**Treg**	• **Markers:** CD4^+^, CD25^+^, CD45RB^+^, FoxP3^+^, CTLA-4^+^, TIM-3^+^
	• **Polarization cytokines:** IL-4		• **Polarization cytokines:** TGF-β
	• **Mechanisms:** Produce cytokines (IL-4, IL-5, IL-6), Increasing antibodies production, Inhibit TH1 function, Promote angiogenesis		• **Mechanisms:** Inhibit cells’ function (CD4^+^ T cells, CD8^+^ T cells, B cells, NK cells)
**M2 macrophage**	• **Markers:** CD163^+^, CD204^+^, CD206^+^	**N2 neutrophil**	• **Markers:** CD66b^+^, CD11b^+^, CD170^high^
	• **Polarization cytokines:** IL-10, TGF-β		• **Polarization cytokines:** TGF-β
	• **Mechanisms:** Produce cytokines (IL-10, IL-13, IL-4, TNF- β, TGF-β), Produce chemokines (CCL17, CCL18, CCL22, CCL24), Increase TH2 and Tregs function, Inhibit CTLs function		• **Mechanisms:** Produce cytokines (VEGF), Produce chemokines (CXCL1, CXCL2, CXCL8, CXCL16, CXCR4, CCL2, CCL3, CCL4, CCL8, CCL12, CCL17), Produce MMP-9, Increase tumor proliferation, Increase angiogenesis, Mediate ECM degradation
**FDC**	• **Markers:** CD23^+^, CD21^+^, CD35^+^	**MDSC**	• **Markers:** **PMN-MDSCs:** CD11b^+^, CD14^−^, CD15^+^, CD66^+^ **M-MDSCs:** Lin^−^, CD11b^+^, CD14^+^, HLA-DR^low^
	• **Polarization cytokines: -**		• **Polarization cytokines: -**
	• **Mechanisms:** Produce mRNA-181a, Decrease Bim levels, Inhibit malignant cell apoptosis		• **Mechanisms:** Produce cytokines (IL-17), Produce inhibitory molecules (Arg1, iNOS, ROS, PGE2), Inhibit cells’ function (CD4 + T cells, CD8 + T cells, and NK cell), Increase Treg function, Increase angiogenesis and metastasis
**MSC**	• **Markers:** CD44^+^, CD166 + , CD105 + , CD90 + , CD45^−^, CD34^−^	**CAF**	• **Markers:** α-SMA^+^, S100A4^+^, FAP^+^, CD10^+^
	• **Polarization cytokines: -**		• **Polarization cytokines: -**
	• **Mechanisms:** Produce cytokines (IL-8, TNF-α, IL-1β, TGF-β), Produce chemokine (CXCL12, CXCL13, CCL19, CCL2), Produce arginase, increase tumor growth, Recruitment monocytes, macrophages, and neutrophils to TME, increase malignant cells homing and adhesion, Transformation to CAFs		• **Mechanisms:** Produce cytokine (IL-8), Produce chemokine (CCL2), Increase tumor growth, increase angiogenesis, Recruitment monocytes, and neutrophils to TME, Decrease neutrophil cel

*NK cell* Natural killer cell, *CTL* Cytotoxic T lymphocyte, *Treg* Regulatory T cell, *FDC* Follicular dendritic cell, *MDSC* Myeloid-derived suppressor cell, *MSC* Mesenchymal stromal cell, *CAF* Cancer-associated fibroblast

Intratumoral T lymphocytes constitute 50% of the total cells within the TME and are categorized based on the expression of CD4 or CD8. Typically, CD4^+^ T lymphocytes support other immune cells, while CD8^+^ cytotoxic T lymphocytes (CTLs) are known to trigger target cell killing by releasing perforin and granzyme B, expression of death ligands and IFN-γ and TNF-α production [53, 54]. CTLs are activated by antigen presentation through MHC-I molecules and the interaction of costimulatory molecules (B7-1 and B7-2) with CD28 [55, 56]. Conversely, inhibitory signals from molecules like CTLA-4, PD-L1/PD-L2, and LAG-3 regulate the activation of CTLs [57, 58]. Clinical studies have shown that higher numbers of intratumoral CD8^+^ T cells are linked to longer overall survival (OS) and disease-specific survival, regardless of other prognostic factors [59]. CD4^+^ T cells play a crucial role in regulating the immune response by boosting Ab production, attracting granulocytes to areas of inflammation, and supporting an efficient immune response by generating cytokines and chemokines [53]. These cells were categorized into effector CD4^+^ T cells, follicular helper T (TFH) cells, and regulatory T cells (Tregs). Effector CD4^+^ T cells polarized to TH1, TH2, and TH 17 cells based on the patterns of various cytokines (See Table 2) [60, 61]. TH1 cells aid the activation of macrophages, NK cells, and CTL, while TH2 cells facilitate humoral immune responses by promoting B-cell growth and antibody production [62–64]. In B-cell NHL, the expression of both TH1 and TH2 cytokines at high mRNA levels has been reported [65]. Traditionally, the TH1 immune response is considered more effective at promoting antitumor immunity, while the TH2 immune response may support tumor growth by promoting angiogenesis and inhibiting the TH1-mediated immune response [66]. Higher levels of IL-4, indicative of a TH2 response, are associated with longer survival, while increased levels of IL-12, a cytokine involved in TH1 immunity, are linked to poorer prognosis in certain NHL types, suggesting that malignant B cells can modulate the effects of TH1 and TH2 cells in different lymphoma types [52, 67]. Besides, defects in TH17 cells have been observed in B cell NHL [52, 68, 69]. TFH cells play specific roles in B-cell clonal selection, maturation, and differentiation into memory cells or plasma cells within germinal centers [70]. TFH cells also facilitate B-cell activation, prevent malignant B cells from undergoing spontaneous apoptosis, and stimulate the proliferation of lymphoma cells [52, 71]. Tregs have a critical role in cancer by restricting immune activation and specific immune responses [72]. In lymphoma biopsy samples, Treg cells are abundant and have been shown to suppress antitumor immunity by inhibiting other intratumoral CD4^+^ and CD8^+^ T-cell populations. TGF-β produced by lymphoma cells can stimulate the expression of FoxP3, a specific marker for Tregs, which can result in the conversion of CD4 + /CD25- T cells into Tregs [73].

In addition to T cells, the immune TME in NHLs also contains tumor-associated macrophages (TAMs), tumor-associated neutrophils (TANs), and myeloid-derived suppressor cells (MDSCs). TAMs are classified to anti-tumorigenic (M1 macrophages) and pro- tumorigenic (M2 macrophages) [74]. M1 macrophages support the growth and differentiation of TH1 cells and NK cells, trigger CTLs cytotoxicity, and mediate ADCC [75–77]. Conversely, M2 macrophages hinder antitumor immunity and promote TH2 and Treg function, as well as CTL suppression [76, 78]. In B-cell lymphoma, TAMs play an important role in tumor progression, drug resistance, and recurrence via multiple mechanisms [78]. Similarly, in the early stages of tumor formation, TANs primarily exhibit the N1 phenotype in the presence of INF-β resulting in CTL activation and recruitment and triggering ADCC. However, as the tumor progresses, TGF-β triggers the transition of TANs to the N2 phenotype. Neutrophils with an N2-like phenotype accompanied by tumor proliferation, blood vessel formation, extracellular matrix (ECM) degradation, and hinder T-cell activation. In addition to their functions, the N1 phenotype and N2 phenotype differ from each other in terms of cell surface markers (See Table 2) [50, 78–81]. MDSCs are a diverse group of cells that include monocytic MDSCs (M-MDSCs) and granulocytic MDSCs (PMN-MDSCs). MDSCs suppress CD4 + T cells, CD8 + T cells, and NK cells through direct cell contact and the production and activation of inhibitory molecules. Furthermore, MDSCs regulate the expansion and activation of Tregs, support tumor angiogenesis and metastasis, and can transform into TAMs at the tumor site. A high prevalence of the M-MDSC subpopulation has been linked to disease progression and decreased OS in B-cell NHL patients [50, 52, 73, 82].

TME also contains stromal cells such as mesenchymal stromal cells (MSCs), which reduce cell death and support tumor growth by secretion of immune molecules. Within the lymphoma TME, MSCs recruit monocytes, macrophages, and neutrophils to the tumor site. Moreover, MSCs can differentiate into cancer-associated fibroblast (CAF)-like cells and secrete diverse chemokines that contribute to the homing and adhesion of lymphoma B cells [52, 83, 84]. CAFs, as another stromal cell, influence a variety of biological processes that advance cancer, including angiogenesis as well as the production and release of growth factors, cytokines, and exosomes. CAFs actively stimulate tumor cell growth, invasion, and inflammation, and contribute to resistance to treatment [50, 52].

Exosomes are an important part of the TME. In lymphomas, exosomes can decrease NK cell-mediated cytotoxicity, trigger immune cell death, and increase treatment resistance through the delivery of various molecules such as interleukins, PGE2, TGF-β, and microRNAs. Additionally, tumor-derived exosomes expedite the activation and growth of MDSCs [50, 52, 85, 86]. Apart from exosomes, chemokines and cytokines present in the TME can also support tumor growth and development. Many studies have shown that the serum level of soluble IL-2Rα, which is produced by CD4^+^ CD25^+^ T cells, is greater in B-cell NHL patients and is associated with a poorer prognosis [73, 87]. The secretion of TGF-β malignant B cells leads to the suppression of TH1 and TH17 cell growth and hinders the proliferation of T cells [73, 88]. Serum IL-10 levels have been demonstrated to be increased in B-cell NHL patients and to be negatively correlated with prognosis [73]. In addition, chemokines released by MSCs in lymphoma, including CXCL13, CCL19/CCL21, and CXCL12, facilitate B-cell adhesion and homing [52].

## NK cell defects in NHL

**Fig. 3:**
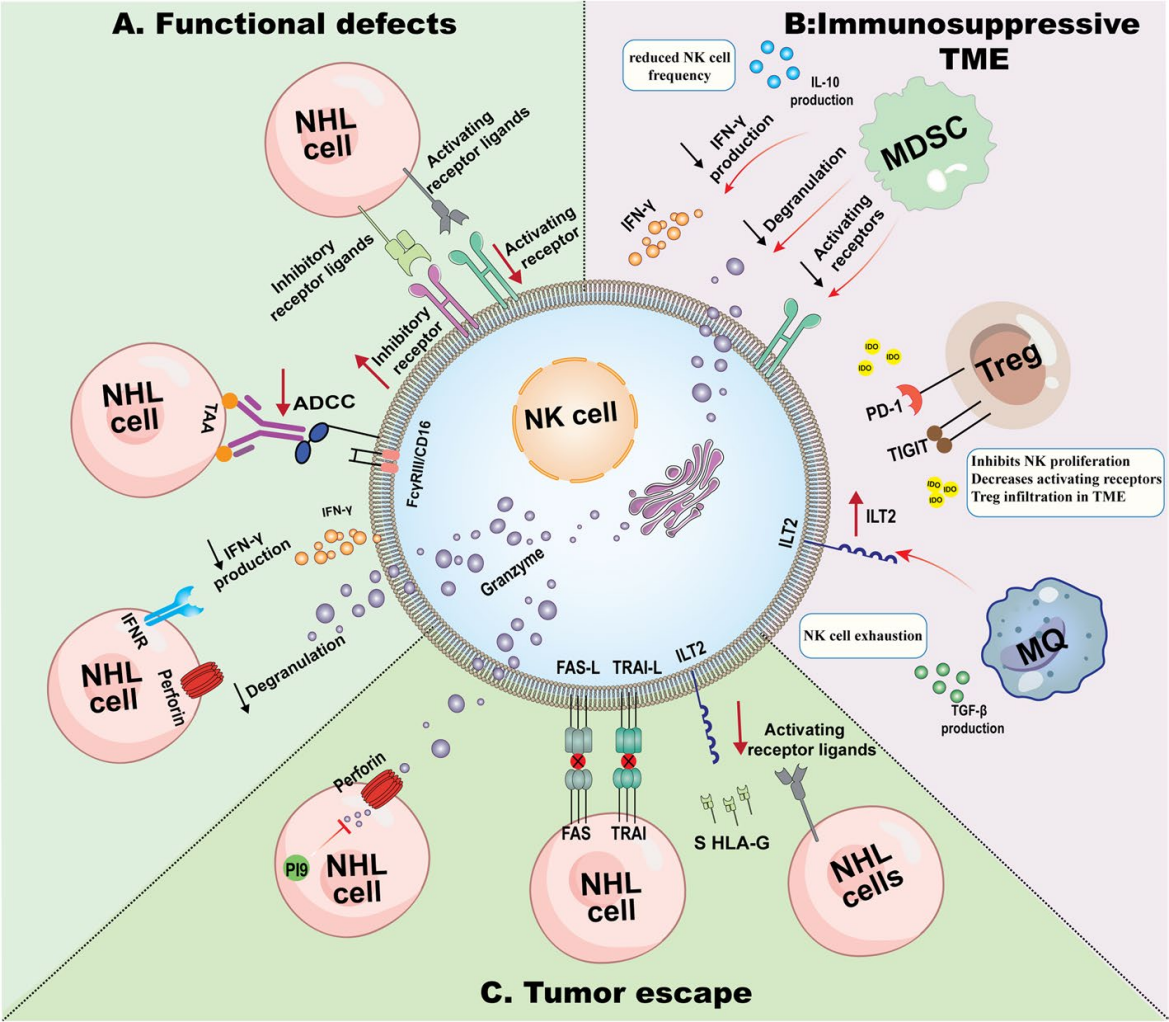
Possible mechanisms of natural killer (NK) cell defects in non-Hodgkin lymphomas (NHLs)**.** The NK cell defects within the TME of NHLs include functional deficiency, the presence of an immunosuppressive TME, and tumor cell escape from NK cell surveillance (as well as quantitative deficiency and distribution abnormalities that are not shown in Fig. 2). See Sect. "NK cell defects in NHL" for more information). A) NK cell functional defects include decreased expression of activating receptors, overexpression of suppressor receptors, decreased ADCC mechanisms, decreased IFN-γ production, and impaired degranulation capacity. B) Immunosuppressive cells such as myeloid-derived suppressor cells (MDSCs), regulatory T cells (Tregs), and M2 macrophages hinder NK cell function through the production of immunosuppressive factors or the expression of inhibitory receptors. C) NHL cells evade NK cell-mediated cytotoxicity via resistance to the perforin/granzyme-mediated apoptosis pathway, resistance to death receptor-mediated apoptosis pathways, and inhibition of NK cell activation

The NK cell defects in the TME of NHL include quantitative deficiency, distribution abnormalities, functional deficiency, the presence of an immunosuppressive TME, and tumor cell escape from NK cell surveillance. Figure 3 summarizes NK cell defects in the TME of NHLs.

### Quantitative deficiency

The absolute NK cell count (A-NKC) decreases in (Diffuse large B cell lymphoma) DLBCL patients. According to Plonquet et al. investigation, one-third of DLBCL patients who present with 2 or 3 adverse prognostic factors of aaIPI have low NKC at diagnosis. Furthermore, they established that NKC was associated with a poorer response to treatment and shorter event-free survival (EFS) [89]. Similarly, flow cytometry analysis of CD3^−^CD56^+^ and/or CD16^+^ cells in the peripheral blood of DLBCL and (follicular lymphoma) FL patients demonstrated that both DLBCL and FL patients had low NKC, which was correlated with a reduction in progression-free survival (PFS) and OS [90]. Furthermore, a deficit in NKC was detected in other NHLs, such as primary central nervous system lymphoma (PCNSL). Lin et al. conducted a study on 161 patients with PCNSL and found that individuals who responded to treatment had a higher median of circulating NKC and NK cell proportion compared to those who did not respond. Their study revealed that PCNSL patients who have a higher baseline NKC display longer OS than those with a low NKC [91]. Consequently, NKC in NHL patients represents a prognostic biomarker for the assessment of clinical outcomes.

### Functional deficiency

Cancer cells have been observed to employ a wide range of mechanisms to escape from the innate immune pressure exerted by NK cells, including abnormalities in NK cytotoxicity function [92]. In the context of NHLs, NK cells have impaired degranulation capacity. According to previous studies, NK cells exhibit defects in the production and exocytosis of cytotoxic granules containing perforin and granzyme [93, 94]. In a protein quantification study involving 12 patients with NHL, it was discovered that while the gene expression levels of perforin and granzyme B were higher in NHL patients compared to the control group, the intracellular levels of perforin in NK cells were lower in NHL patients [93]. Furthermore, a separate study conducted by Baychelier et al. found that patients who developed NHL after undergoing lung transplantation exhibited an accumulation of NK cells with low expression of perforin and impaired degranulation against NHL target cells [94]. Other aspects of NK cell hypofunction include decreased cytokines production, e.g. IFN-γ and TNF-α, the overexpression of suppressor receptors such as T-cell immunoreceptors with immunoglobulin and ITIM domains (TIGIT), and the decreased expression of activating receptors such as TIM-3 [95]. It should be noted that TIGIT has been associated with NK cell exhaustion [96]. In addition, decreased CD16 expression in NK cells and impaired ADCC activity were observed in newly diagnosed and refractory NHL patients [95, 97].

NK cells in refractory NHL exhibit downregulated expression of activating receptors, including NKp30, NKp46, and NKG2D. Further investigation revealed that de novo NHL development was correlated with increased NKG2A and CD62L expression but reduced inhibitory KIR and CD57 receptor expression [94]. Essa et al. demonstrated that DLBCL patients with advanced stages of the disease have significantly lower NKp44 levels than patients with earlier stages of DLBCL. This decrease in NKp44 may be attributed to the high level of IL6 and TGF-β in the advanced stages of the disease, which in turn downregulate NK activating receptors [98]. The expression of CD16 and NKG2D activating receptors on the surface of CD56^dim^ cells was also reported to be decreased after rituximab treatment [99] (Fig. 3A).

### Immunosuppressive TME

Successful interaction between NK cells and dendritic cells (DCs) and the production of chemokines are required to induce effective antitumor immunity by NK cells (Fig. 1). This process is negatively affected by TME, especially cellular and soluble components of the TME, which are associated with the escape of cancer cells due to the lack of effective immune responses [100]. Several immune suppressive cells, like MDSC, TAMs, and Tregs negatively interfere with NK cell activation and function. In a phase 2 clinical trial conducted by Bachanova et al., the frequencies of MDSCs and Tregs were investigated about adoptive NK cell therapy response in patients with NHL. Results from the trial indicated that patients who exhibited higher frequencies of MDSCs and Tregs, along with the adoptive NK cells, had a poorer response to therapy [95]. Similarly, Sato et al. demonstrated that the accumulation of MDSCs leads to NK cell depletion in NHL patients [101]. Increased numbers of MDSCs were reported in DLBCL, MZL, MCL, high‐grade B‐cell lymphoma (HGBL), PCNSL, and FL. Interestingly, MDSCs are markedly increased in high-grade NHLs and may be a potential prognostic marker [102]. The inhibitory effect of MDSCs on NKG2D expression and IFN-γ production in NK cells was confirmed in both in vivo and in vitro experiments [103]. The downregulation of other types of NK cell activating receptors, such as NKp30 and NKp46, was also detected [104, 105]. Further analysis in a murine lymphoma model revealed that MDSCs, which can secrete IL-10, reduced the frequency of NK cells [101]. Additionally, the coculture of MDSCs with NK cells has been shown to negatively affect the degranulation capacity of these cells through the TIGIT/CD155 pathway [105].

Tregs are another immunosuppressive cell type that limits adoptive NK cell therapy in NHL patients. Increased numbers of Tregs expressing high levels of Foxp3 following high-dose chemotherapy and IL2 administration before adaptive NK cell infusion interfere with NK cell expansion [106]. Treg infiltration in the TME could be justified via Indoleamine-2,3-dioxygenase (IDO). IDO is an immunosuppressive enzyme that catalyzes the conversion of tryptophan to kynurenine [107]. NHL patients who overexpress IDO simultaneously exhibit increased levels of FoxP3, a Treg marker [108]. In addition, IDO not only inhibits NK cell proliferation but also decreases activating receptors [107]. In a study conducted by Ninomiya et al., it was found that 32% of DLBCL patients exhibit overexpression of IDO, which is associated with unfavorable clinical outcomes [109]. Additionally, Yoshikawa et al. reported elevated levels of tryptophan-derived kynurenine in DLBCL patients [110].

Crosstalk between M2 macrophages and NK cells is another barrier to NK cell function in the TME. M2 macrophages limit NK cells' function by triggering the expression of inhibitory receptors immunoglobulin-like transcript 2 (ILT2/ CD85j), an NK cell inhibitory receptor [111]. In NHL, a high density of M2 macrophages in DLBCL of the central nervous system (CNS-DLBCL) has been detected and accounted for poor clinical outcomes [15].

Among other immunosuppressive factors, TGF-β has been investigated in NHL, and previous studies revealed TGF-β signaling Dysregulation in mantle cell lymphoma (MCL), FL, and DLBCL. The TGF-β signaling cascade is dysregulated through various mechanisms, such as altered receptor expression, disrupted SMAD signaling, and disturbances in epigenetic and genetic processes [112]. TGF-β inhibits IFN-γ expression, affects the metabolic pathway of NK cells, and reduces NKG2D and NKp30 expression, which are essential for tumor cell recognition and elimination, as well as for the effective interaction between natural NK cells and DCs [113, 114]. Interestingly, MDSCs and M2 macrophages participate in NK cell exhaustion by producing TGF-β1 (Fig. 3B) [103, 115].

### Evasion mechanism

#### Resistance to apoptosis

Tumor cells in NHLs may escape from NK cell-mediated cytotoxicity through resistance to perforin/granzyme-mediated apoptosis. For this purpose, tumor cells may exhibit elevated intrinsic levels of proteinase inhibitor 9 (PI9), which functions to restrict the proteolytic action of granzyme B and secure their survival [116]. In this line, Bladergroen et al. verified that P19 was overexpressed in different types of T/B-NHL, such as extranodal T-cell NHL, enteropathy type T-cell NHL, NK/T-cell nasal-type lymphoma, and DLBCL [116]. Furthermore, cancer cells may escape apoptosis by inactivating apoptotic pathways activated by death receptors. The death ligands FasL/CD95L and TRAIL, which are members of the TNF family, are expressed in NK cells. These ligands interact with their respective receptors, Fas/CD95 and TRAIL-R, present on the surface of target cells. Upon interaction, the death domain (DD) is activated, initiating the apoptotic signaling cascade and ultimately leading to apoptosis [117]. According to previous studies, loss of Fas/CD95 expression was found in some FL and diffuse B/T-cell lymphomas [118], mucosa-associated lymphoid tissue lymphomas (MALTLs) [119], and cutaneous B-cell lymphomas (CBCLs) [120], which are associated with poor prognosis. In addition, mutations in Fas/CD95 have been reported in GC-derived B-cell lymphomas, such as primary nodal DLBCL, MALT-type lymphomas, FL, and anaplastic large cell lymphoma (ALCL) [121, 122]. A somatic mutation in TRAIL-R, which is correlated with the loss of chromosome 8p21-22, has also been detected in NHLs (Fig. 3C) [123].

#### Inhibition of NK cell activation

CD58 or lymphocyte-function antigen 3 (LFA-3) is known as an NK cell activator that interacts with CD2 on the NK cell surface [124–126]. Based on this speculation, mutations or deletions in CD58 also prevent NK cell function, which has been reported in DLBCL and FL [125, 126]. HLA-G is an inhibitory molecule in both membrane-bound and soluble isoforms that suppresses NK cells through interaction with its ILT2 [127]. The serum level of soluble HLA-G increased in NLHs such as DLBCL, FL, and peripheral T-cell lymphoma, which may disrupt NK cell function and be involved in lymphoma development [128]. Notably, HLA-G expression in lymphoma is a double-edged sword with protective and destructive effects [129]. To explore the evasion mechanisms, strategies that disturb NK cell receptors are also considered. The investigation by Satwani et al. revealed that, incubation of NHL cells with romidepsin enhanced NK cell cytotoxicity. Subsequently, they reported that romidepsin increases the surface expression of the NKG2D ligands MIC A/B on lymphoma cells. Based on the results of this study, impairment of NK cell function may be related to decreased expression of activating receptor ligands such as MIC A/B [130]. The immune checkpoints PD-1 and PD-L1 also restrict NK cell function, and PD-1/PD-L1 axis blockade unleashes NK cell cytotoxicity [127]. Research conducted by Laurent et al. revealed that DLBCL cells exhibit notably elevated levels of PD-1 and PD-L1/2 compared to FL cells. Notably, some DLBCL tumor cells coexpress both PD-1 and PD-L1/2. Interestingly, there are more PD-L1/2-positive lymphoma cells in the activated B-cell (ABC) subtype of DLBCL (ABC-DLBCL) than in the GC subtype (GC-DLBCL) [129]. Similarly, Kiyasu et al. reported that PL-1 is frequently expressed in tumor cells in DLBCL and is associated with poor prognosis [131].

## NK cell immunotherapy in NHLs

In NHL, the A-NKC of the autograft directly influences clinical outcomes of following HSCT [132]. In a randomized, double-blind phase III clinical trial, patients with NHL who received an autograft with an A-NKC ≥ 0.5 × 10^9^ cells/kg demonstrated 5-year OS and 5-year PFS rates of 87% and 71%, respectively. In contrast, patients infused with an autograft A-NKC < 0.5 × 10^9^ cells/kg experienced 5-year OS rates of 55% and 5-year PFS rates of 32% [133]. With a 10.6-year median follow-up in the final update, the 13-year OS rates demonstrated a significant difference between groups, with a rate of 46% for the cohort infused with autograft A-NKC ≥ 0.09 × 10^9^ cells/kg compared to 36% for the group infused with A-NKC < 0.09 × 10^9^ cells/kg (*P*-value < 0.02) [134]. Faster and robust recovery of NK cells following HSCT is another factor that can affect clinical outcomes [135]. Porrata et al. reported that NHL patients with an A-NKC ≥ 80 cells/µL on day 15 after autologous HSCT had longer OS and PFS than patients with lower counts (not reached vs 5 months, p0.001; not reached vs 3 months, p0.0001, respectively) [136]. These findings suggest that the early post-HSCT recovery of NK cells may play a crucial antitumor role in the potential graft-versus-tumor (GVT) effect, given that NK cells are the only immune effector cells that reach normal numbers and function post-HSCT [137].

Several early studies have employed the administration of low-dose subcutaneous rlL-2 to promote the recovery and cytotoxic activity of NK cells as an effective approach to eradicate residual disease and prevent relapse following autologous HSCT in NHL patients [138, 139]. In a clinical trial involving patients with R/R high-grade NHL, researchers demonstrated that the administration of a low dose of rlL2 early after autologous HSCT for a duration of one year is well-tolerated and leads to the in vivo expansion of CD16^+^/CD56^+^ NK cells. Significantly, compared to their baseline quantity and function before starting treatment, the expanded CD56^bright^ NK cell subsets exhibited enhanced activity against K562 cells (an NK-sensitive cell line) and CD16-mediated redirected killing activity against P815 target cells (an NK-resistant cell line). All ten patients who participated in the trial remained free from relapse for a period ranging from 5 to 34 months (median 16 months) after initiating rIL2 therapy. Notably, two patients who still had residual disease following HSCT experienced complete disease disappearance after rIL2 treatment [138]. Building upon the encouraging outcomes of these early studies, clinical trials have explored the adoptive transfer of ex vivo activated autologous NK cells or lymphokine-activated killer cells as a therapeutic approach for patients with lymphoma [140, 141]. The adoptive transfer of autologous NK cells was found to be feasible and safe, although only a limited antitumor effect was observed [142]. This limitation primarily stemmed from the matching of inhibitory receptors on autologous NK cells with self-MHC class I present on tumor cells, leading to "self" recognition signals that dampen NK cell activation and subsequent antitumor effects [142]. Furthermore, the adoptive transfer of autologous NK cells is costly and frequently requires multiple apheresis procedures, and the dose of injected NK cells is limited to approximately 10^7^/kg [143]. To overcome these limitations, researchers have recently used allogeneic NK cells for lymphoma immunotherapy. In the phase 1 clinical trial conducted by Green Cross LabCell Corporation, the safety and possible efficacy of allogeneic NK cells were assessed in patients with malignant lymphoma or advanced solid tumors. In this study, allogeneic NK cells (namely, MG 4101) were obtained from random healthy unrelated donors and expanded in culture bags supplemented with IL-2, irradiated autologous feeder cells, and OKT3. Multiple doses of MG4101 were administered in the dose range of 1 × 10^6^ cells/kg to 3 × 10^7^ cells/kg without any signs of GVHD or serious toxicity. Among the 17 evaluable patients, only 8 exhibited stable disease (SD), while the disease progressed in the remaining patients. The median PFS for patients with SD was 4 months, ranging from 2 to 18 months [144]. The results of this study indicated that the use of alloreactive NK cells alone was not sufficient to eliminate the disease mass completely. As a result, researchers explored the combination of NK cells with other strategies to enhance their therapeutic effectiveness in subsequent studies [135].

### NK cells combined with mAbs

Over the past two decades, the therapeutic effects of at least 570 monoclonal antibodies (mAbs) have been investigated in clinical trials. Among them, 79 therapeutic mAbs, including 30 mAbs for the treatment of hematological malignancies, have received approval from the United States food and drug administration (FDA) and are currently commercially available [145]. When mAbs bind to their targets, they can kill cancer cells through a variety of mechanisms, including programmed cell death (PCD), complement-dependent cytotoxicity (CDC), and ADCC [146]. Among these mechanisms, ADCC is an effective immune mechanism that is triggered when therapeutic mAbs are employed to eliminate cancer cells [147]. During the ADCC process, the FC region of the antibody is ligated to its corresponding FC receptor (FcR) on the plasma membrane of immune effector cells, while the Fab portion of the antibody attaches to target antigens on the surface of the cancer cell [148]. Human NK cells serve as crucial effector cells in the context of ADCC by expressing CD16A, which is a low-affinity receptor for IgG1 and IgG3 antibodies [149]. Given the likelihood that the efficacy of ADCC-mediated tumor cell elimination relies on the ratio of effector to target cells, the number and function of NK cells have been investigated as potential biomarkers to predict the response to anti-CD20 immunotherapy in NHL patients [90, 150]. Klanova et al. reported that low peripheral blood NKC in FL and DLBCL patients receiving anti-CD20 mAbs (rituximab or obinutuzumab) plus chemotherapy were linked to shorter PFS in both FL and DLBCL patients and diminished OS specifically in FL patients [90] Hence, the number of NK cells in individuals with lymphoma is important for determining their prognosis [151].

The administration of adoptive NK cells to enhance the ADCC capabilities of mAbs is a growing area of intervention that has been explored in recent years [152]. There are several ongoing and completed clinical trials exploring the safety and effectiveness of combining mAbs with infusions of autologous or allogeneic NK cells in patients with NHL (Tables 3 and 4). In a recent phase I study, Tanaka et al. investigated the infusion of ex vivo-expanded autologous NK cells in combination with rituximab-containing chemotherapy in patients with relapsed CD20^+^ malignant lymphoma [153]. Expanded autologous NK cells with high expression of NKp30, NKp44 and CD16 were intravenously infused (up to 10 × 10^6^ cells/kg) into lymphoma patients one day after rituximab-combined salvage chemotherapy. The combination was safe and feasible, and among the nine lymphoma patients, seven achieved complete response (CR), with a median duration of 44 months (range: 6–56 months). However, it is difficult to determine the precise contribution of autologous NK cells to the clinical response, given that chemotherapy was administered to eight of nine patients after NK cell infusion [153]. In another study on chemotherapy-refractory NHL patients, allogeneic NK cell therapy (dose of 0.5–3.27 × 10^7^ cells/kg) in combination with IL-2 and rituximab was found to be safe and effective in 4 of 15 evaluable patients, with 2 patients achieving CR lasting 3 and 9 months and 2 patients obtaining partial response (PR) [95]. Moreover, in a recent phase I study employing ex vivo-expanded allogeneic NK cells (namely, MG4101) plus rituximab after lymphodepleting chemotherapy for R/R NHL patients, Yoon et al. demonstrated that the treatment was well tolerated and led to a PR in 4 patients and a CR in 1 patient, yielding an overall response rate (ORR) of 55.6% [154]. Notably, one patient achieved a lasting CR that extended beyond 806 days [154].

**Table 3:** Ongoing clinical trials of NK cell therapies for NHL

#	NCT Number	Phase/N	Trial status	Study Design	Conditions	Interventions	Doses	Cells source	Outcome Measures	Sponsor/Location
1	NCT03019666	Phase I/39	Completed	Nonrandomized, parallel assignment, open label	MM and NHLs	Cyclophosphamide + fludarabine + NAM-NK + elotuzumab (for MM) or rituximab (for NHL) + IL-2	Not disclosed	PB	Primary: safety Secondary: occurrence of TRM, occurrence of disease response and number of patients alive without progression	Masonic cancer center, university of Minnesota/USA
2	NCT02259348	Phase II/12	Terminated	Single group assignment, open label	ALL, AML, CML, JMML, MDS and NHL	Cyclophosphamide + fludarabine + IL-2 + melphalan + thiotepa + rituximab + NK cells + T-cell depleted HPCT + CD45RA-depleted HPCT + G-CSF	Not disclosed	Not disclosed	Primary: percentage of participants engrafted by day 42 posttransplant Secondary: incidence of malignant relapse, EFS, OS, incidence and severity of aGVHD and cGVHD and rate of TRM	St. Jude children's research hospital/USA
3	NCT02280525	Phase I/8	Completed	Nonrandomized, single group assignment, open label	Leukemia and lymphoma	Lenalidomide + fludarabine and cyclophosphamide for CLL and low-grade lymphoma + rituximab (for patients with B-cell cancer) + NK cells	1 × 10^7^ cells/kg	UCB	Primary: MTD of NK cells with lenalidomide and lymphodepleting chemotherapy Secondary: response rate of NK cells with lenalidomide and lymphodepleting chemotherapy determined by BMA/BMB	M.D. Anderson cancer center/USA
4	NCT01181258	Phase II/16	Completed	Nonrandomized, single group assignment, open label	NHL and CLL	rituximab, fludarabine, cyclophosphamide, methylprednisolone + NK cells + IL2	1.5 to 8 × 10^7^ cells/kg	PB	Primary: number of patients with a PR or CR Secondary: SAEs, time to disease progression and patients with expansion of NK cells	Masonic cancer center, university of Minnesota/USA
5	NCT00625729	Phase I, II/6	Terminated	Single group assignment, open label	Relapsed NHL and CLL	Fludarabine + cyclophosphamide + rituximab + NK cells + aldesleukin (rIL-2) + G-CSF	1.5 to 8 × 10^7^ cells/kg	PB	Primary: NK cell expansion Secondary: number of patients with IL-15 production and NK cell expansion, OR, number of patients whose disease progressed after treatment, number of patients with adequate NK cells infused and OS	Masonic cancer center, university of Minnesota/USA
6	NCT00383994	Phase I/, 6	Completed	Single group assignment, open label	B-cell lymphoid malignancies	GM-CSF + rituximab + NK cell	Not disclosed	Not disclosed	Primary: DLT for NK cells infusions	M.D. Anderson cancer center/USA
7	NCT03019640	Phase II/22	Completed	Single group assignment, open label	R/R B-Cell NHL	Carmustine + etoposide + cytarabine + melphalan + lenalidomide + rituximab (for patients who are CD20^+^) + NK cell + HSCT + filgrastim	Not disclosed	UCB	Primary: TRM30 Secondary: number of participants who survived on day 180	M.D. Anderson cancer center/USA
8	NCT02843061	Phase I, II/30	Completed	Randomized, parallel assignment, open label	Recurrent B-cell lymphoma	Rituximab + NK cells	Each time 10 billion cells, 4 times in all	Not disclosed	Primary: relief degree evaluated by the RECIST Secondary: PFS and OS	Fuda cancer hospital, Guangzhou/China
9	NCT04074746	Phase I, II/30	Active, not recruiting	Single group assignment, open label	R/R CD30^+^ HL or NHL	Fludarabine + cyclophosphamide + AFM13-NK + AFM13	Not disclosed	UCB	Primary: SAEs Secondary: OS, EFS, ORR, duration of response and immune reconstitution studies	M.D. Anderson Cancer Center/USA
10	NCT02890758	Phase I/14	Completed	Nonrandomized, single group assignment, open label	NHL, AML, MDS, ALL, CLL, CML, …	NK cell infusion + ALT803	Dose escalation from 1 × 10^6^ to 5 × 10^7^ cells/kg	Not disclosed	Primary: MTD and number of patients without GVHD Secondary: number of patients with HR, patients’ response for radiographically measurable lesions, patients with malignant lymphoma response, patients’ response for WM, CL, MM, CML, average duration of response, average duration of OS, RFS and in vivo NK levels	Brenda Cooper /USA
11	NCT01386619	Phase I, II/15	Completed	Single group assignment, open label	AML, MDS, lymphoma, NB and RMS	HSCT + NK DLI	> 1 × 10^7^ NK cells/kg	PB	Primary: feasibility and safety Secondary: efficacy	University hospital, Basel, Switzerland/Germany and Switzerland
12	NCT01287104	Phase I/34	Completed	Sequential assignment, open label	NHL. AML, ALL, CML and HD	Stem cell + NK cell	1 × 10^5^ to 1 × 10^7^ cells/kg	PB	Primary: number of patients who received 2 doses of NK cell infusions and number of patients who received the highest dose level of NK cells Secondary: number of participants with mild, moderate and/or severe cGVHD, DFS, OS since date of transplant, occurrences of viral infection and/or reactivation, decline in IL-7 and IL-15, cell numbers posttransplant, number of participants with presence of KIR gene mismatch, SAEs and non-SAE	National cancer institute/USA
13	NCT00990717	Phase I/8	Completed	Single group assignment, open label	NHL, HL, leukemia	Irradiated NK-92 cells	Level I: 1 × 10^9^ cells/m^2^ Level II: 3 × 10^9^ cells/m^2^ Level III: 5 × 10^9^ cells/m^2^	NK-92	Primary: DLT Secondary: efficacy, immune response directed against the NK-92 cells and kinetics of NK92 cells	University health network, Toronto/Canada
14	NCT00823524	Phase I, II/47	Completed	Single group assignment, open label	Lymphoma, leukemia, MDS, myeloma, brain and CNS tumors and solid tumors	HSCT + NK cell	Not disclosed	PB	Primary: safety Secondary: efficacy	Asan medical center/ South Korea
15	NCT00789776	Phase I, II/41	Completed	Single group assignment, open label	NHL, ALL, AML, MDS, HL, MM, CLL, CML	Fludarabine + cyclophosphamide + TBI + HSCT + NK cells + mycophenolate + tacrolimus	Not disclosed	PB	Primary: DLT, number of participants with relapsed disease and aGVHD, number of nonrelapse participant mortalities, number of participants who experienced graft failure Secondary: number of subjects surviving posttransplant and number of participants who experienced cGVHD	Fred Hutchinson cancer center/USA
16	NCT00697671	Phase I/48	Completed	Nonrandomized, parallel assignment, open label	NHL, MDS, CML, JMML,	Clofarabine + cyclophosphamide + etoposide + NK cell + IL-2	Not disclosed	PB	Primary: Safety Secondary: efficacy and NK cell persistence, phenotype and function of NK cells after infusion	St. Jude children's research hospital/USA
17	NCT00660166	Phase I/13	Completed	Nonrandomized, single group assignment, open label	NHL, HL, MM, AML	AHSCT + NK cell	Not disclosed	PB	Primary: safety Secondary: duration of donor NK cells in the recipient's blood, patient survival at 100 days and at one year post treatment, occurrence of new cancer during the first-year post infusion and systemic infections during the first 30 days post infusion	Tufts medical center/USA
18	NCT00586703	Phase I/21	Completed	Single group assignment, open label	Lymphoma	Nonmyeloablative ASCT + NK cell	Up to 1 × 10^7^ CD56^+^ cells/kg	PB	Primary: safety Secondary: efficacy of the regimen in terms of OS	David Rizzieri, MD/USA
19	NCT01619761	Phase I/13	Unknown	Nonrandomized, parallel assignment, open label	NHL, HL, ALL, AML, MDS, CML, CLL, Myeloma	Lenalidomide + fludarabine + melphalan + rituximab (for CD20 positive patients) + NK cells + UCB-HSC + tacrolimus + mycophenolate	Not disclosed	UCB	Primary: generation of a minimum of 5 × 10^6^ NK/kg cells in at least 60% of patients, treatment-related mortality and incidence of SAEs Secondary: proportion of patients with aGVHD and cGVHD, OS, DFS, time to initial platelet recovery and time to initial absolute neutrophil count recovery	M.D. Anderson cancer center/USA
20	NCT04673617	Phase I, II/108	Recruiting	Nonrandomized, sequential assignment, open label	NHL	Cyclophosphamide + fludarabine + rituximab + bendamustine + NKcell + IL-2	Not disclosed	UCB	Primary: safety and tolerability, ORR, identify the R2PD and efficacy	Artiva biotherapeutics, Inc/USA
21	NCT03778619	Phase I, II/9	Unknown	Single group assignment, open label	R/R NHL	Fludarabine + cyclophosphamide + NK cells (MG4101) + IL-2	Group 1: 1 × 10^7^cells/Kg Group 2: 3 × 10^7^cells/Kg Group 3: 9 × 10^7^cells/Kg	PB	Primary: at phase I MTD and at phase II ORR Secondary: at phase I: ORR at phase II: CR, PR, OS, time to progression and time to response	GC cell corporation/South Korea
22	NCT04023071	Phase I/72	Terminated	Nonrandomized, parallel assignment, open label	B-cell lymphoma and AML	Cyclophosphamide + fludarabine + obinutuzumab or rituximab (for B-cell lymphoma) + NK cell (FT516) + IL-2	Not disclosed	iPSC	Primary: DLT and incidence, nature and severity of AEs Secondary: anti-tumor activity of FT516 and FT516 pharmacokinetic data	Fate therapeutics/USA

*NAM-NK* Nicotinamide expanded-natural killer, *IL* Interleukin, *PB* Peripheral blood, *UCB* Umbilical cord blood, *iPSC* Induced pluripotent stem cell, *TRM* Treatment related mortality, *HSCT* Hematopoietic stem cell transplantation, *ASCT* Autologous Stem Cell Transplant, *G-CSF* Granulocyte colony-stimulating factor, *AE* Adverse event, *SAEs* Serious Adverse Events, *MTD* Maximum tolerated dose, *BMA/BMB* Bone marrow aspiration/Bone marrow biopsy, *OR* Overall response, *ORR* Overall response rate, *OS* Overall survival, *EFS* Event-Free Survival, *PFS* Progression-free survival, *aGVHD* Acute graft-versus-host disease, *CR* Complete response, *PR* Partial response, *RFS* Relapse free survival, *DFS* Disease-free Survival, *cGVHD* Chronic graft versus host disease, *DLT* Dose limiting toxicities, *TRM30* Treatment-related mortality within 30 days, *RECIST* Response evaluation criteria in solid tumors, *HR* Hematological response, *WM* Waldenstrom's macroglobulinemia, *CL* Cutaneous lymphomas, *MM* Multiple myeloma, *CML* Chronic myeloid leukemia, *ALL* Acute lymphoblastic leukemia, *AML* Acute myeloid leukemia, *MDS* Myelodysplastic syndrome, *JMML* Juvenile myelomonocytic leukemia, *NHL* Non-Hodgkin lymphoma, *HL* Hodgkin lymphoma, *CLL* Chronic lymphocytic leukemia, *NB* Neuroblastoma, *RMS* Rhabdomyosarcoma, *R2PD* Recommended phase 2 dos, *KIR* Killer-cell immunoglobulin-like receptors, *DLI* Donor lymphocyte infusion, *CNS* Central nervous system

**Table 4:** Outcome of clinical trials of NK cells in NHL patients

#	Study	Conditions/n	Cells source	Culture methos	Final product Characteristics	Infused dose of NK cells	Chemotherapy regimen before NK cell infusion	Combination therapy	Adverse event/Toxicity	Clinical response	Reference
1	Phase I, II (NCT04673617)	R/R NHL including DLBCL (*n* = 9), FL (*n* = 5), MCL (*n* = 2), LPL/WM (*n* = 1)	UCB	Not disclosed	Not disclosed	1 × 10^9^ cell/dose or 4 × 10^9^ cell/dose	Cyclophosphamide and fludarabine	Rituximab	Grade 1 CRS in 2 patients, grade 1 fever in 1 patient, infections in 3 patients, PD in 3 patients	67% (4/6) ORR in rituximab cohort, 27% (3/11) ORR in monotherapy (NK cells) cohort	Khanal et al. [155]
2	Phase II (NCT01181258)	DLBCL (*n* = 11), MCL (*n* = 2), FL (*n* = 1), CLL (1), LPL/WM (*n* = 1)	PB	CD3/CD19-depleted cells were cultured in X-VIVO media supplemented with 10% human AB serum and 1000 U/mL IL-2 for 16–18 h	Viability > 70%, NK cell content ≥ 20%, T-cell content ≤ 3.0 × 10^5^/kg, B-cell content < 3% and endotoxin < 5EU/kg	0.5–3.27 × 10^7^/kg in association with IL-2	Initially, the regimen consisted of pentostatin, cyclophosphamide, and denileukin diftitox, but it was later revised to include fludarabine, cyclophosphamide, and methylprednisolone	Rituximab	No symptoms or signs of GVHD, neurotoxicity, or persistent marrow aplasia were reported	Of 14 (29%) evaluable patients, 4 had (29%) objective responses (including 2 CR and 2 PR) at two months	Bachanova et al. [95]
3	phase I (NCT03019666)	R/R NHL including DLBCL (*n* = 9), FL (*n* = 10), MCL (*n* = 1)	PB	CD3 depleted cells were culture for 14 days with IL-15 (20 ng/ml) and NAM (5 mM)	Viability > 97.46%, mean T-cell content 0.7%, mean NK cell content 96.12% and mean B-cell content 0.58%	Three dose level: 2 × 10^7^, 1 × 10^8^ or 2 × 10^8^ cells/kg in association with IL-2	Cyclophosphamide and fludarabine	Rituximab	There was no evidence of CRS, neurotoxicity, GVHD, or marrow aplasia	Of 19 evaluable patients, 11 (58%) had CR and 3 (16%) had PR for an ORR of 74%. PFS at 1 and 2 years was estimated at 50% and 35%, respectively. The OS at 2 years was 73%	Cichocki et al. [156]
4	phase I UMIN000014072	R/R CD20^+^ lymphoma including DLBCL (*n* = 5), FL (*n* = 4)	PB	The PBMC were culture for 3 weeks in SCGM media supplemented with IL-15 (10 ng/mL), IL-2 (5 ng/mL), anti-CD3 mAb (10–1,000 ng/mL), tacrolimus (0.1 ng/mL) and dalteparin sodium (5–10 U/mL)	The final products contained 80.5% CD56^+^ CD3^–^ NK cells and 26.2 ± 11.6% of CD56^+^ CD3^+^ NKT cells	1 to 10 × 10^6^cells/kg	Not disclosed	Rituximab	There was no AEs related to NK cells infusion	Of 9 patients, seven (78%) patients achieved CR with a median duration of 44 months	Tanaka et al. [153]
5	phase I NCT03778619	R/R NHL including DLBCL (*n* = 6), MCL (*n* = 2) and MZL (*n* = 1)	PB	CD3 depleted cells were culture in CellGro SCGM media with 1% autoplasma, irradiated autologous PBMCs, anti-CD3 mAb (10 ng/mL) and IL2 (500 IU/mL)	Viability > 90% with CD16^+^CD56^+^ NK cells > 95%,	level 1:1 × 10^7^, level 2: 3 × 10^7^, level 3: 9 × 10^7^ cells/kg in association with IL-2	Cyclophosphamide and fludarabine	Rituximab	CRS in 1 patient. GVHD, neurotoxicity, and DLTs were not observed	Of 9 patients, 4 (44%) patients achieved PR, one (11%) achieved CR and 4 (44%) showed PD. The median duration of response was 45 days	Yoon et al. [154]
6	phase II NCT03019640	R/R NHL including DLBCL (*n* = 16), MCL (*n* = 2) and FL (*n* = 1)	UCB	CB mononuclear cells were plated in RPMI media with irradiated aAPC feeder cells and IL-2 (100IU). On day 7, cultured cells were CD3-depleted and remaining cells were then culture in the same conditions for an additional 7 days	viability > 96% purity > 98% median CD3 CD16^+^CD56^+^ cells 98.91%	10^8^ cells/kg	Carmustine, etoposide, cytarabine, melphalan and lenalidomide	Rituximab	GVHD, neurotoxicity, and DLTs were not observed	At median follow-up of 47 months, the RFS and OS rates were 53% and 74%, respectively	Nieto et al. [157]
7	phase I NCT04023071	R/R BCL including DLBCL (*n* = 10), FL (*n* = 2) and MZL (*n* = 1)	iPSC	iPSCs transduced and differentiated in CD34^+^ cells hnCD16^+^ for 18– 21 days, then CD34^+^ cells culture in B0 medium supplemented with 20% human serum and NK cell initiating cytokines (IL-3, IL-7, IL-15, SCF, Flt3L) on EL08-1D2 stroma (culture days 28–35)	Not disclosed	dose cohort 1: 3 × 10^7^ dose cohorts 2: 9 × 10^7^ dose cohorts 3: 30 × 10^7^cells/dose	Cyclophosphamide and fludarabine	Rituximab or obinutuzumab	No CRS, ICANS, or GVHD of any grade were reported	Of 11 patients, 8 (73%) patients achieved an objective response (including 6 CR and 2 PR) and 3 (27%) showed PD	Patel et al. [158]
8	Phase I, II NCT04074746	CD30^+^ lymphomas including HL (*n* = 37) and NHL (*n* = 5)	UCB	CB-derived NK cells were first activated with IL-12/IL-15/IL-18-and then expanded with K562 feeder cells expressing mbIL-21, 4-1BBL and CD48 and exogenous IL-2 for 14 days. Finally, NK cell complexed with AFM13	Viability > 95% NK cells bound to AFM13 > 90% NK cell content > 94% T-cell content 0.02%	level 1:10^6^ level 2: 10^7^ level 3: 10^8^ NK cells/kg	Cyclophosphamide and fludarabine	AFM13	No cases of CRS, ICANS or GVHD of any grade were reported	ORR 92.8% and CR 66.7% at median follow-up of 14 months, the EFS/OS rates are 31%/76%; median EFS/OS were 8 months	Nieto et al. [159]
9	Phase I, II NCT00625729	Relapsed NHL or CLL (*n* = 6)	PB	CD3 depleted cells were activated by IL-2 overnight	Not disclosed	1.5–8 × 10^7^ cells/kg in association with IL-2	Cyclophosphamide and fludarabine	Rituximab	Not disclosed	Of 6 patients, 4 (67%) patients had OR at 3 months, while 2 (33%) had PD at 6 months. OS at 6 months was 3/6 patients	Mamo et al. [160, 161]

*R/R* Refractory/relapsed, *NHL* Non-Hodgkin lymphoma, *HL* Hodgkin lymphoma, *CLL* Chronic lymphocytic leukemia, *DLBCL* Diffuse large B-cell lymphoma, *FL* Follicular Lymphoma, *MZL* Marginal zone lymphomas, *MCL* Mantle cell lymphoma, *WM/LPL* Waldenström macroglobulinemia/lymphoplasmacytic lymphoma, *PB* Peripheral blood, *UCB* Umbilical cord blood, *iPSC* Induced pluripotent stem cell, *ORR* Overall response rate, *PD* Progression disease, *CRS* Cytokine release syndrome, *ICANS* Immune associated neurological symptom, *GVHD* Graft-versus-host disease, *CR* Complete response, *OS* Overall survival, *PR* Partial response, *PFS* Progression-free survival, *EFS* Event-Free Survival, *RFS* Relapse free survival, *NAM* Nicotinamide, *mAb* Monoclonal antibody, *DLTs* Dose limiting toxicities, *aAPC* Artificial antigen presenting cells, *IL* Interleukin, *SCF* Stem cell factor, *FLT3L* FMS-like tyrosine kinase 3 ligand, *mb* Membrane bound

In addition to autologous or allogeneic peripheral blood (PB)-derived NK cells, an increasing number of clinical trials have scrutinized the safety and efficacy of other NK cell sources, including cord blood (CB) [6, 155, 157], induced pluripotent stem cells (iPSCs) [158], and immortalized NK cell lines [162], for NHL immunotherapy. For example, in a phase 1/2 clinical trial, the safety and clinical activity of AB-101 (an allogeneic, nongenetically modified, CB-NK cell product) has been evaluated as a monotherapy and combined with rituximab for the treatment of R/R NHL patients [155]. The results from this study indicated that the concurrent administration of both agents was safe, resulting in an ORR of 67% in 6 patients (CR observed in 3 patients and PR in 1 patient), in contrast to an ORR of 27% in cohorts receiving AB-101 alone [155]. Another study by Katayoun Rezvani’s group assessed the efficacy of ex vivo-expanded CB-NKs in combination with rituximab and high-dose chemotherapy in NHL patients who were candidates for autologous HSCT [157]. Patients received rituximab and high-dose chemotherapy from days 13 through 7, lenalidomide from days 7 through 2, and CB-NK cells (10^8^/kg) on day 5 before to autologous HSCT. CB-NK cells were detectable in vivo for two weeks, regardless of their HLA mismatch status. Importantly, no adverse events attributable to the CB-NK cells were observed. At a median follow-up of 47 months, the rates of relapse free survival (RFS) and OS were 53% and 74%, respectively [157].

NK-92 is an immortalized IL-2-dependent CD16^−^ NK cell line that was isolated and successfully established by Klingman et al. in 1992 from a patient suffering from lymphoma. NK-92 cells exhibit potent cytotoxicity against several cancer cells, a phenomenon primarily ascribed to the overexpression of numerous activating receptors, concurrent downregulation of almost all inhibitory receptors, and heightened expression of perforin and granzyme. Furthermore, NK-92 cells can continuously proliferate with a doubling time of 2–4 days, are easily obtainable, and have a homogeneous phenotype [163, 164]. However, due to their cancerous nature, NK-92 cells must be mitotically inactivated prior to infusion into patients to inhibit undesired clonal proliferation, which restricts their persistence and expansion in vivo, and allogeneic administration demands very high doses of NK-92 cells [165]. In 2008, Arai et al. demonstrated for the first time the feasibility and safety of administering NK-92 cells (up to 3 × 10^9^) to cancer patients [166]. Recently, a phase I dose-escalation study using NK-92 cells (1 × 10^9^ cells/m^2^, 3 × 10^9^ cells/m^2^ and 5 × 10^9^ cells/m^2^) for refractory hematological malignancies that relapsed after autologous HSCT was conducted by Williams et al. [162]. A total of 12 patients were enrolled in this trial, including 2 patients with HL and 5 patients with NHL. The infusions of irradiated NK-92 cells were well-tolerated even at high doses and resulted in CR in one HL patient and a minor response (defined as 10–30% regression of target tumor lesions without the occurrence of new lesions or progression of nontarget lesions) in 2 NHL patients. Notably, in this study, no NK-92 cells were detected more than 15 min after infusion [162]. As mentioned earlier, NK-92 cells are highly dependent on exogenous IL-2 for survival and lack the CD16 receptor, thus impeding their capacity to mediate ADCC [167]. To address this, NK-92 cells have been modified to internally express IL-2 and the high-affinity CD16 receptor [168, 169]. Currently, this product, designated high-affinity NK (haNK), is being investigated in several clinical trials for solid tumors [170, 171]. Furthermore, preclinical data indicated that the combination of haNK cells withmAbs, such as daratumumab for multiple myeloma (MM) and rituximab for NHL, may have a synergistic effect. However, further clinical investigation is required to validate these approaches for NHL [163].

**Fig. 4:**
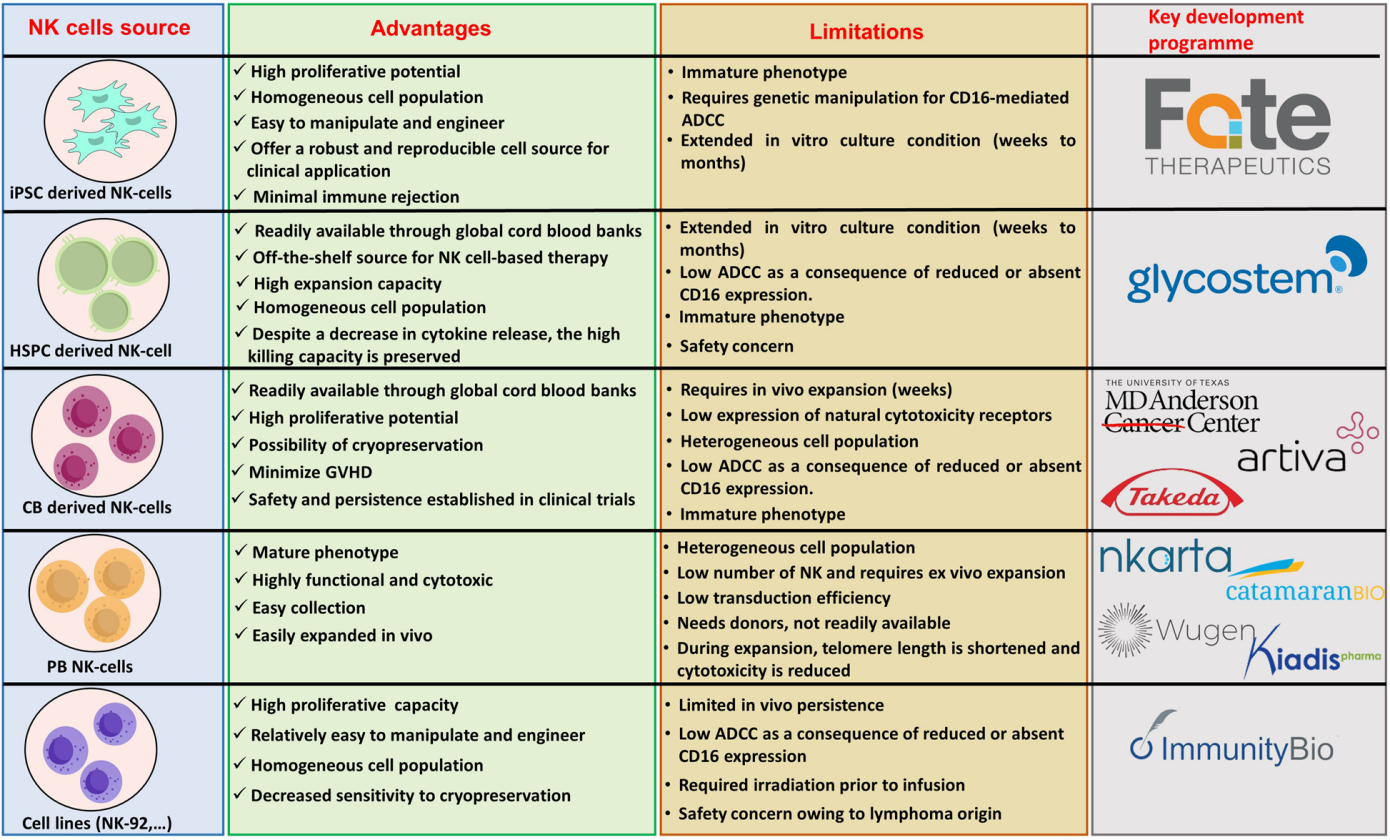
Overview of the advantages and limitations of different sources of NK cells

NK cells derived from iPSCs (iNKs) are another promising avenue for NK cell therapy and have the potential to address challenges commonly encountered with other sources of NK cells (Fig. 4) [172]. To generate iNK cells, somatic cells are first differentiated into iPSCs and then into CD34^+^ hematopoietic stem and progenitor cells (HSPCs). Subsequently, NK cells differentiate from HSPCs using cytokines (IL-3, IL-7, IL-15, SCF, and FLT3L) or stromal-based feeder cell lines and are then cocultured with feeder cells for further expansion [173, 174]. Currently, iPSC-based NK cell platforms have been evaluated in several clinical trials as monotherapies or in combination with mAbs for the treatment of hematological malignancies or solid tumors [158, 175–177]. As an example, FT516 is an iPSC-derived NK cell product modified to express high-affinity, cleavage-resistant Fc receptor (CD16A), with a preliminary report of 18 patients with R/R B-cell lymphoma in combination with rituximab demonstrating safety, with no evidence of GVHD, ICANS or CRS. Patients received two cycles of treatment consisting of a conditioning regimen (fludarabine and cyclophosphamide, each for 3 days), a single dose of rituximab and three weekly cycles of FT516 (four patients received 90 million cells/dose, seven patients received 300 million cells/dose, and seven patients received 900 million cells/dose) accompanied by IL-2 (6 MIU after each dose of FT516). Of the 18 patients, 10 patients were naive to treatment with autologous CD19-targeted CAR-T cells, and eight patients were previously treated with autologous CD19-targeted CAR-T-cell therapy. A total of 8/10 naive patients achieved an ORR (including 5 patients who achieved CR), and 3/8 patients previously treated with CD19-targeted CAR-T-cell therapy achieved an OR and CR [158, 177].

### NK cells combined with bispecific antibodies

Bispecific killer engagers (BiKEs) were created with the intention of having one "arm" that binds to CD16 on NK cells and the other "arm" that targets a specific antigen on tumor cells [178]. The engager serves as a replacement for traditional antibody-Fc interactions in facilitating the immunological synapse between tumor cells and NK cells, thereby promoting NK activation and the killing of tumor cells [179]. Therefore, the use of BiKEs could enhance the function of NK cells by creating a stronger interaction when binding to anti-CD16 compared to the interaction between CD16 and the natural Fc portion of antibodies [180]. Moreover, BiKEs are nonimmunogenic and have rapid clearance properties, making them easy to engineer to target known tumor antigens. In addition to these advantages, BiKEs may offer advantages over mAbs due to their smaller size, which allows for better distribution in the body. This approach is especially beneficial for treating solid tumors [181–183]. Currently, several clinical trials are being conducted to evaluate the effectiveness of BiKEs in combination with NK cell therapy as a treatment for patients with lymphoma. Some of these trials focused on AFM13 [159, 184]. AFM13 is a tetravalent, bispecific innate cell engager that targets CD16A/CD30 and activates innate immune cells such as NK cells and macrophages [185]. AFM13 acts as a mediator by binding to CD16A on NK cells and to CD30 on lymphoma cells, which aids in the recruitment and activation of NK cells in proximity to tumor cells [186]. AFM13 was initially tested as a single therapy in a phase 1 clinical study for patients with R/R lymphoma [187]. The study showed that AFM13 treatment was safe and well-tolerated and led to positive tumor responses in several patients [187]. CB-NK cells precomplexed with AFM13 were recently tested within an ongoing phase I/II clinical trial for patients with refractory CD30-positive lymphomas. Forty-two patients (37 patients with HL and 5 patients with NHL with a median of seven prior lines of therapy) received fludarabine/cyclophosphamide followed by CB-NK cells precomplexed with AFM13 and three weekly IV infusions of AFM13. The results of this study showed that AFM13 in combination with NK cells was safe for patients with no instances of CRS, ICANS, or GVHD and resulted in an ORR of 92.8% and a CR rate of 66.7%. All four patients who had previously failed CD30 CAR-T-cell therapy achieved a CR [159].

### NK cells combined with *CAR* structure

**Fig. 5:**
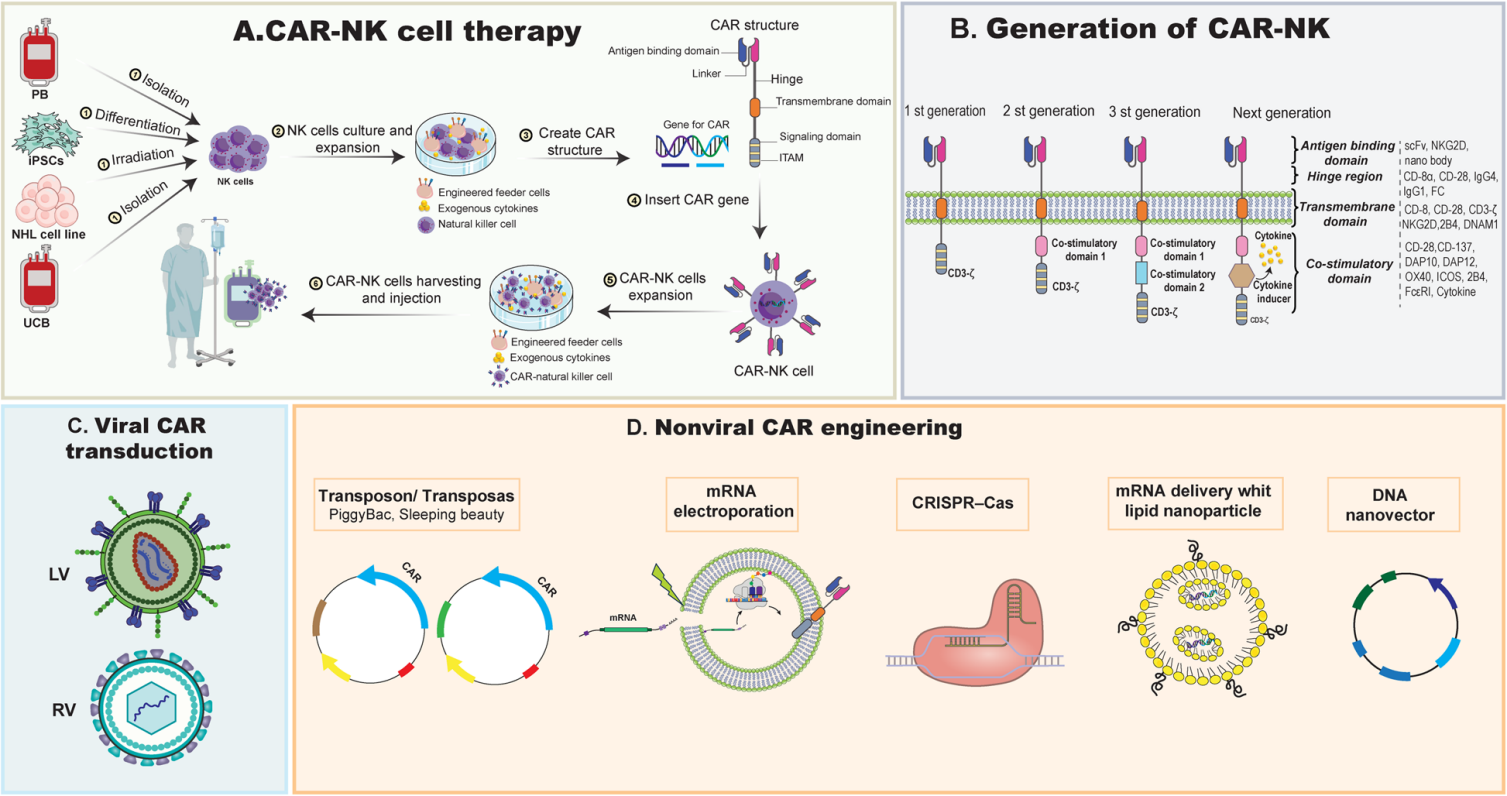
An overview of (A) CAR-NK cell therapy workflow in NHLs, (B) CAR structure and generation and (C, D) various methods of delivering CAR into NK cells

The CAR construct plays a crucial role in activating cells that have been transduced with CAR. The CARs employed in CAR-NK cells are often analogous to those utilized in CAR-T cells. A CAR consists of four essential components: an extracellular binding domain, a hinge region, a transmembrane domain, and one or more intracellular signaling domains (Fig. 5). Single-chain antibody variable fragments (scFvs) originate from a tumor-specific antibody and have the ability to bind to a particular antigen displayed on the surface of cancer cells. Moreover, the intracellular signaling domains are obtained from the cytoplasmic domains of ITAMs found in TCRs or other stimulating receptors [188]. The extracellular binding domain of CAR-modified effector cells enhances specificity by targeting tumor-associated antigens (TAAs). The hinge region serves as a connection between the extracellular binding domain and the transmembrane domain. The intracellular signaling domains in different generations of CARs possess different compositions, which affects the potency of the activation signal transmitted and consequently influences the cytotoxic capability against tumor cells (Fig. 5) [189]. The first generation of CARs consisted of only the CD3-ζ activation signaling domain. Subsequent generations of CARs incorporated one or two supplementary costimulatory molecules, including CD28, ICOS, 4-1BB, CD27, OX40, and CD40. CD28 and 4-1BB are the predominant molecules utilized among this group of molecules [190, 191]. Researchers have utilized other molecules as activation signaling domains for NK cells, in addition to the commonly used CARs that are applicable for both CAR-T cells and CAR-NK cells. CD244 (2B4), a member of the signaling lymphocyte activation molecule (SLAM) family, can also serve as a costimulatory molecule. The overexpression of 2B4 in NK cells leads to an enhanced ability to amplify signals and increased innate cytotoxicity against tumor cells [192]. DAP-12 is present on NK cells and plays a role in transmitting signals through the NK-activating receptors NKG2C and NKp44. Additionally, DAP-10 is involved in signal transmission through NKG2D [193, 194]. Hence, DAP-12 and DAP-10 can transmit intracellular signals in CAR-NK cells. In addition, NK cells modified with DAP-12-based CARs exhibited superior performance compared to that of NK cells modified with CD3-ζ-based CARs [193]. Recent research has indicated that NKG2D ligands are overexpressed in several hematological malignancies. Hence, the NKG2D-DAP-10-CD3-ζ CAR, which specifically targets NKG2D ligands, holds significant promise for the treatment of blood malignancies [195].

Transduction of the CAR gene into NK cells encompasses viral transduction, namely, retrovirus-based and lentivirus-based approaches, as well as transfection techniques such as electroporation, lipofection, and their combination with transposon systems (Fig. 5) [11]. CAR constructs are commonly integrated into a retrovirus or lentivirus-based expression vector. These vectors are then used to transduce primary NK cells or NK cell lines, with NK-92 being the most frequently used. The transduction of retroviral vectors shows a high level of effectiveness (ranging from 43 to 93%) in primary NK cells. However, the occurrence of insertional mutagenesis and its negative consequences significantly limit the use of this method in clinical applications [196]. However, lentivirus-based transduction is considered to be a safer method. Although its transduction efficiency in peripheral blood mononuclear cell (PBMC)-derived NK cells ranges from 8 to 16%, there is still an opportunity for improvement [197]. RNA transfection methods are economical strategies that have greater efficacy in transferring genes. However, the production of CAR constructs using this method is temporary, lasting for approximately 3–5 days. Although the short therapeutic time frame is a limitation, the temporary nature of CAR therapy may lower the occurrence of CAR-associated adverse effects, such as on-target off-tumor effects [195, 197, 198]. The integration of DNA into cells using transposon systems, such as PiggyBac (PB) and sleeping beauty (SB), in combination with transfection methods has emerged as an appealing strategy for generating cells that express transgenes in a safer and more stable manner [199, 200]. The SB transposon vector has proven to be a cost-effective and efficient means of gene transfer. However, its suitability for use with CAR-NK cells has not yet been evaluated [201].

CAR-NK cells are safer than CAR-T cells. The enhanced safety of CAR-NK cells can be attributed to two primary factors. CRS and neurotoxicity are frequently adverse effects of CAR-T-cell therapy [202]. The cytokine storm triggered by CAR-T cells, specifically TNF-α, is primarily facilitated by proinflammatory cytokines such as IL-1 and IL-6 [203]. CAR-NK cells secrete a variety of cytokines, such as IFN-γ and GM-CSF, which differ from the cytokines produced by CAR-T cells. Second, CAR-T cells can cause life-threatening GVHD due to HLA limitations. On the other hand, NK cells, which are considered important cells that initiate the GVT response early on, can potentially prevent GVHD by eliminating recipient antigen-presenting cells and CTLs [204]. Furthermore, CAR-NK cells may exhibit superior effectiveness in targeting and destroying cancerous cells compared to CAR-T cells. CAR-NK cells possess the ability to identify and execute their cytotoxic functions via both their designed and innate killing capabilities. By utilizing CARs, effector cells can enhance their ability to selectively target and eliminate a specific antigen with greater efficiency. Unlike CAR-T cells, CAR-NK cells retain the inherent ability of NK cells to destroy target tumor cells even when the expression of specific tumor antigens is reduced [205]. Moreover, the production of CAR-NK cells is more convenient than that of CAR-T cells. Due to the absence of the risk of GVHD, NK cells can be obtained from either a donor who is a match or a donor who has an HLA mismatch, hence expanding the pool of potential donors and enhancing the overall quality of the end products [206].

Recently, CAR-NK cell therapy has been assessed in various clinical trials for the treatment of lymphoma (Tables 5 and 6). CB-CAR-NK cells are presently employed in a clinical trial at MD Anderson Cancer Center, specifically targeting CD19 cells, and yielding highly favorable outcomes. 37 patients with R/R CD19-positive malignancies were enrolled in this trial and treated with CB-CAR-NK cells in two phases: a dose-escalation phase and an expansion phase. In the dose-escalation phase (*n* = 11), patients received a conditioning regimen (fludarabine and cyclophosphamide, each for 3 consecutive days) followed by the infusion of CB-CAR-NK cells (three patients received 10 × 10^4^ cells/kg, four patients received 10 × 10^5^ cells/kg, and four patients received 10 × 10^6^ cells/kg). In the expansion phase (*n* = 26), patients were first treated with 10 × 10^6^ cells/kg CB-CAR-NK. Then, the trial was amended to include a second expansion cohort in which patients received a single flat dose of 8 × 10^8^ cells/kg CB-CAR-NK. A retroviral vector including an anti-CD19-CD28-CD3-ζ CAR, an IL-15 gene, and a suicide switch was utilized for transduction. None of the patients developed neurotoxicity or GVHD, and only one patient developed mild CRS (grade I). The ORR (including PR and CR) on days 30 and 100 for the 37 patients was 48.6%. The 1-year OS and PFS were 68% and 32%, respectively. Compared with non-responders, patients who achieved OR had higher levels and longer persistence of CB-CAR-NK cells [6, 207].

**Table 5:** Ongoing clinical trials of CAR-NK cell therapies for NHL

#	**NCT Number**	**Phase/N**	**Trial status**	**Study Design**	**Conditions**	**Antigen**	**Vector**	**Interventions**	**Doses**	**Cells source**	**CAR construct**	**Outcome Measures**	**Sponsor/Location**
1	NCT05472558	Phase I/48	Recruiting	Single group assignment, open label	R/R B-cell NHL	CD-19	Lentiviral vector	Anti-CD19 CAR-NK	2.5 × 10^8^ cells, 5 × 10^8^ cells, 1 × 10^9^ cells	UCB	Not disclosed	Primary: safety, tolerability and determine the recommended dosage of anti-CD19 CAR-NK cell Secondary: CR, PFS, DOR, OS, PR, ORR and immunogenicity of anti-CD19 CAR-NK cell	Zhejiang university/China
2	NCT03056339	Phase I, II/44	Completed	Single group assignment, open label	R/R B-lymphoid malignancies (ALL, CLL, NHL)	CD-19	Retroviral vector	Fludarabine + cyclophosphamide + Mesna + CAR-NK Cells + AP1903 (in case of GVHD or CRS after CAR-NK infusion)	Not disclosed	UCB	CD19-CD28-zeta-2A-iCasp9-IL15	Primary: safety and efficacy Secondary: ORR (CR and PRR), determining the persistence of injected cells and comprehensive immune reconstitution studies	M.D. Anderson cancer center/USA
3	NCT03824964	Phase I/10	Unknow	Single group assignment, open label	Refractory B-Cell lymphoma	Dual-target CD19/22	Not disclosed	Anti-CD19/CD22 CAR-NK cells	50 to 600 × 10^3^ cells/kg	Not disclosed	CD19/CD22-CD-244	Primary: occurrence of treatment related AEs	Allife medical science and technology Co., Ltd./China
4	NCT03692767	Phase I/9	Unknow	Sequential assignment, open label	R/R B-cell lymphoma	CD-22	Not disclosed	Anti-CD22 CAR-NK cells	50 to 600 × 10^3^ cells/kg	Not disclosed	CD22-CD-244	Primary: occurrence of treatment related AEs	Allife medical science and technology Co., Ltd./China
5	NCT03690310	phase I /9	Unknown	Single group assignment, open label	R/R B-cell lymphoma	CD-19	Not disclosed	Anti-CD19 CAR NK cells	50 to 600 × 10^3^ cells/kg	NK-92 cell line	CD19-CD-244	Primary: occurrence of treatment related AEs	Allife medical science and technology Co., Ltd./China
6	NCT04639739	phase I /9	Unknown	Single group assignment, open label	R/​R NHL	CD-19	Not disclosed	Fludarabine + cyclophosphamide + anti-CD19 CAR NK cells	2 × 10^6^/kg 6 × 10^6^/kg 2 × 10^7^/kg	Not disclosed	Not disclosed	Primary: DLTs and incidence and severity of AEs and SAEs Secondary: ORR, PFS and OS	Xinqiao hospital of Chongqing/China
7	NCT04796675	Phase I/27	Recruiting	Single group assignment, open label	B Lymphoid Malignancies (ALL, CLL and NHL)	CD-19	Retroviral vector	Fludarabine + cyclophosphamide + Anti-CD19 CAR-NK cells	0.01 × 10^7^ 0.1 × 10^7^ 1.0 × 10^7^/kg	UCB	Not disclosed	Primary: occurrence of treatment related AEs Secondary: ORR, CR, PFS, DOR and OS	Wuhan union hospital/China
8	NCT04887012	Phase I/25	Recruiting	Single group assignment, open label	R/R B-cell NHL	CD-19	Lentiviral vector	Anti-CD19 CAR-NK cells	Not disclosed	PB	Not disclosed	Primary: DLTs and ORR Secondary: OS, PFS, pharmacokinetics of CAR positive cells and pharmacokinetics of CAR-NK cells	Second affiliated hospital, school of medicine, Zhejiang university Hangzhou, Zhejiang/China
9	NCT05020678	Phase I/150	Recruiting	Single Group assignment, open Label	B-cell cancer (NHL, ALL, CLL)	CD-19	Retroviral vectors	Fludarabine + cyclophosphamide + anti- CD19CAR-NK cells (combination cohorts will additionally receive rituximab with each cycle)	3 × 10^8^ NK cells (6 × 10^6^/kg for patients < 50 kg)	PB	CD19-CD134 (OX40)-CD3z-IL-15	Primary: occurrence of TEAEs, proportion of subjects experiencing DLTs and ORR Secondary: assessment of CAR-NK half-life, duration of persistence, host immune response against CAR-NK and ORR	Nkarta Inc/USA
10	NCT05379647	Phase I/24	Recruiting	Non-randomized, parallel assignment, open label	B-Cell Malignancies (r/r ALL and lymphoma)	CD-19	Not disclosed	Cyclophosphamide + Fludarabine + VP-16 + CAR-NK cell as Monotherapy (for patients with r/r B-ALL) or in combination with rituximab (for patients with r/r B-cell lymphoma)	Not disclosed	iPSC	Not disclosed	Primary: incidence of TEAEs and proportion of subjects experiencing DLTs Secondary: ORR, DOR, PFS, OS, pharmacokinetics of CAR-NK cells and EFS	Zhejiang university/China
11	NCT05618925	Phase I/20	Not yet recruiting	Randomized, single group assignment, pen label	R/R NHL	CD-19	Not disclosed	lymphodepleting chemotherapy + CAR-NK cell in combination with rituximab (cohort A) or in combination with rituximab and N-803 (cohort B)	Not disclosed	NK-92 cell line	Not disclosed	Primary: safety Secondary: ORR	ImmunityBio, Inc/USA
12	NCT05570188	Phase I, II	Withdrawn (the principal investigator decides to stop)	Single group assignment, open label	B-Cell malignancies	CD-19	Not disclosed	HSC + CAR-NK cell	Dose level 1: 5–10 × 10^6^/kg, Dose level 2: 1–2 × 10^7^/kg, Dose level 3: 2–5 × 10^7^/kg	Not disclosed	Not disclosed	Primary: incidence of AEs Secondary: granulocyte implantation time, platelet implantation time, red blood cell implantation time, DOR, DCR, OS, and PFS	Kunming hope of health hospital/China
13	NCT03579927	Phase I, II	Withdrawn (lack of funding)	Single group assignment, open Label	B-cell NHL	CD-19	Retroviral vector	Rituximab + carmustine, etoposide, cytarabine and melphalan + CAR-NK cell + ASCT + filgrastim	Not disclosed	UCB	CD19-CD28-z-2A-iCasp9-IL15	Primary: incidence of AEs, CR or PR Secondary: PFS, OS, response status and number of CAR-NK cells in blood	M.D. Anderson cancer center/USA
14	NCT02892695	Phase I, II/10	Unknown	Single group assignment, open label	CD19^+^ Lymphoma (NHL)	CD-19	Not disclosed	Anti-CD19 CAR-NK cells	Not disclosed	NK-92	CAR.19-CD28- 41BB-CD3ζ	Primary: AEs Secondary: ORR	Person gen biotherapeutics Co., Ltd/China
15	NCT05092451	Phase I, II/94	Recruiting	Randomized, single group assignment, open label	R/R Hematologic Malignances (AML/MDS and B-Cell lymphoma)	CD-70	Not disclosed	Cyclophosphamide + fludarabine phosphate + anti- CD70 CAR-NK	Not disclosed	UCB	CAR.CD70-IL15 (full construct not disclosed)	Primary: number of participants with TEAEs, CR, PR and number of Participants who are alive and in remission	MD Anderson cancer center/USA
16	NCT05667155	Phase I/48	Recruiting	Single group assignment, open label	B-cell NHL	Dual-target CD19/70	Lentiviral vector	Anti-CD19/70 CAR-NK	Not disclosed	UCB	CD19/CD70-IL-15 (full construct not disclosed)	Primary: incidence of DLTs Secondary: OS, ORR, DOR, CR, PR and PFS	Second affiliated hospital, school of medicine, Zhejiang university/China
17	NCT05654038	Phase I, II/30	Recruiting	Single group assignment, open label	B-Cell hematologic malignancies	CD-19	Not disclosed	HSCT + Anti-CD19 CAR-NK cells	Dose level 1: 5 -10 × 10^6^/kg; dose level 2:1–2 × 10^7^/kg; dose level 3:2–5 × 10^7^/kg	Not disclosed	Not disclosed	Primary: incidence of AEs Secondary: Granulocyte implantation time, platelet implantation time, red blood cell implantation time, DOR, DCR, OS and PFS	920th hospital of joint logistics support force of people's liberation army of China/China
18	NCT05020015	Phase II/242	Recruiting	Nonrandomized, parallel assignment, open label	R/R B-cell NHL	CD-19	Retroviral vector	Fludarabine + cyclophosphamide + CD19 CAR-NK cells (TAK-007)	200 × 10^6^ to 800 × 10^6^ TAK-007	UCB	Not disclosed	Primary: incidence of AEs, number of participants with clinically significant changes in laboratory parameters, number of participants with clinically significant changes in vital signs and ORR Secondary: ORR, CR, DOR, PFS, OS, number of participants with AEs	Takeda/USA
19	NCT05842707	Phase I, II/48	Recruiting	Single group assignment, open label	R/R B-cell NHL	Dual-target CD19/CD70	Not disclosed	Dual Anti-19/70 CAR-NK cell	Not disclosed	UCB	Not disclosed	Primary: DLTs Secondary: ORR, OS, CR, DOR, PFS	Aibin Liang/China
20	NCT05673447	Early phase I/12	Recruiting	single group assignment, open label	R/R DLBCL	CD-19	Not disclosed	Anti-CD19 CAR-NK cells	6 × 10^8^ cells, 1 × 10^9^ cells, 1.5 × 10^9^ cells	Not disclosed	Not disclosed	Primary: incidence of DLTs and TEAEs Secondary: ORR, OS, CR and DCR	Changhai hospital/China
21	NCT05645601	Phase I/12	Recruiting	Single group assignment, open label	R/R B-cell malignancies	CD-19	Not disclosed	Fludarabine, + cyclophosphamide + CD19-CAR-NK	5 × 10^6^cells/kg, 2 × 10^7^/kg	Not disclosed	Not disclosed	Primary: DLTs, TEAEs and ORR Secondary: PFS, OS and proportion of subjects with MRD negative response	Affiliated hospital to academy of military medical sciences/China
22	NCT05410041	Phase I/15	Recruiting	single group assignment, open label	R/R B-cell malignancies (ALL, CLL and NHL)	CD-19	Not disclosed	Fludarabine + cyclophosphamide + CD19-CAR-NK	1–3 × 10^7^cells/kg	Not disclosed	Not disclosed	Primary: safety and ORR Secondary: concentration of CAR^+^ NK cells in PB, pharmacodynamic data in PB Other outcome measures: DOR, PFS and OS	Beijing Boren hospital/China
23	NCT05336409	Phase I/75	Recruiting	Nonrandomized, sequential assignment, open label	CD19^+^ B-cell malignancies	CD-19	Not disclosed	Lymphodepleting chemotherapy + CD19-CAR-NK (CNTY-101) + IL-2	Not disclosed	iPSC	CD19 CAR-CD28-CD3zeta-IL-15-expressing	Primary: MTD and RP2R Secondary: CR, OR, DOR, TTR, PFS, OS, plasma concentration for cnty-101, Tmax, terminal disposition phase half-life for 101, AUC0, percentage of participants with at least one TEAE, percentage of participants with clinically significant laboratory abnormalities and time to treatment initiation	Century therapeutics, Inc/USA
24	NCT05739227	Early phase I/12	Recruiting	single group assignment, open label	R/R B-cell hematologic malignancies (ALL, CLL and B-cell Lymphoma)	CD-19	Not disclosed	Fludarabine + cyclophosphamide + etoposide + CD19-CAR-NK (JD001)	1 × 10^6^cells/kg, 5 × 10^6^cells/kg, 2 × 10^7^cells/kg	PB	Not disclosed	Primary: DLT and ORR Secondary: PFS, OS, MRD-ORR	Xuzhou medical university/China
25	NCT06206902	Phase I/56	Recruiting	Single group assignment, open label	R/R NHL	Not disclosed	Not disclosed	Cyclophosphamide + CAR-NK Cells (F01)	0.5–3 × 10^9^ CAR-NK Cells	Not disclosed	Not disclosed	Primary: safety Secondary: ORR, DOR, PFS, OS, Cmax of F01 cells, Tmax of F01 cells, AUC0-last of F01 cells, Clast of F01 cells, Tlast of F01 cells, Dynamic changes of cytokine levels, Anti-CAR and IL-15 antibodies, PB lymphocyte subsets, detection rate of replicable virus	Shanghai Simnova Biotechnology Co. Ltd/China

*CAR* Chimeric antigen receptor, *RP2R* Recommended phase 2 regimen, *TEAE* Treatment emergent adverse event, *MRD-ORR* Minimal-residual disease negative overall response rate, *Cmax* Gene copy number amplification, *Tmax* Time to reach the maximum concentration, *AUC0* The area under the drug curve, *Clast* Last detectable concentration point, *Tlast* Time of the last detectable concentration, *ALL* Acute lymphoblastic leukemia, *AML* Acute myeloid leukemia, *MDS* Myelodysplastic syndrome, *DLCBL* Diffuse large B-cell lymphoma, *NHL* Non-Hodgkin lymphoma, *HD* Hodgkin disease, *CLL* Chronic lymphocytic leukemia, *CR* Complete response, *PR* Partial response, *RFS* Relapse free survival, *DLT* Dose limiting toxicities, *AEs* Adverse events, *SAEs* Serious adverse events, *TTR* time-to-response, *DSR* disease control rate, *DFS* Disease-free Survival, *ORR* Overall response rate, *OS* Overall survival, *DOR* Duration of response, *PFS* Progression-free survival, *DCR* Disease control rate, *PB* Peripheral blood, *UCB* Umbilical cord blood, *iPSC* Induced pluripotent stem cell, *IL* Interleukin, *HSCT* Hematopoietic stem cell transplantation, *ASCT* Autologous Stem cell transplantation, *CRS* Cytokine release syndrome, *GVHD* Graft-versus-host disease

**Table 6:** Outcome of clinical trials of CAR-NK cells in NHL patients

#	Study	Conditions/n	source	Product name	CAR-structure	Vector/Target	Infused dose	Chemotherapy regimen	Combination therapy	Adverse event/Toxicity	Clinical response	Reference
1	Phase I NCT05020678	DLBCL (*n* = 6), FL (*n* = 6), MCL (*n* = 1) and MZL (*n* = 1)	PB	NKX019	Anti-CD19.OX40.CD3z.IL-15	Retroviral vector/CD-19	Dose level 1: 1.5 × 10^9^ Dose level 2: 1 × 10^9^ Dose level 3: 3 × 10^8^ cells	Cyclophosphamide and fludarabine	None	No patients developed signs of CRS, ICANS and GVHD	Of 14 patients, 8 (57%) achieved CR and 4 (29%) showed PD. Of the 8 patients who achieved CR, 3 patients subsequently relapsed, each after more than 6 months of remission	Dickinson et al. [208]
2	Case report NCT05336409	R/R FL	iPSC	CNTY-101	Anti-CD19.CD28.CD3zeta-IL-15. Additionally, CNTY-101 product engineered to avoid recognition by patient CD8 + T, CD4 + T and NK cells. Knockout of β2 M, designed to prevent CD8 + T-cell recognition, knock-out of the CIITA, designed to prevent CD4 + T-cell recognition, and knock-in of the HLA-E gene, designed to enable higher expression of the HLA-E protein to prevent killing of CNTY-101 cells by host NK cells. Furthermore, to potentially improve safety, the CNTY-101 cells were engineered with an EGFR safety switch	Not disclosed/CD-19	1 × 10^8^ cells	Cyclophosphamide and fludarabine	None	No CRS or neurotoxicity were detected	Ongoing CR of a duration of 5 months since the first CNTY-101 infusion	Ramachandran et al. [209]
3	Phase I NCT04245722	R/R BCL (*n* = 26)	iPSC	FT596	Anti-CD19.NKG2D TM.2B4.CD3z.IL-15RF	Not disclosed/CD-19	3 × 10^7^ cells 9 × 10^7^ cells 30 × 10^7^ cells	Cyclophosphamide and fludarabine	Rituximab or obinutuzumab	No GvHD and ICANS were observed. Two cases of CRS were reported	Overall: 18/26 patients (69%) achieved ORR, including 12/18 patients (46%) achieved CR on day 29 following a single dose of FT596. Combination therapy (comprising a total of 12 patients): 9/12 patients (75%) achieved ORR including 7 patients (58%) with CR on day 29 following a single dose of FT596	Bachanova et al. [210]
4	Phase I, II NCT03056339	DLBCL (*n* = 17), FL (*n* = 4), MCL (*n* = 1), MZL (*n* = 2), CLL (*n* = 6), CLL-RT (5), ALL (*n* = 1) and LPL (*n* = 1)	UCB	Not applicable	Anti-CD19. CD28 + IC.CD3z.IL-15. Casp9	Retroviral vector/CD-19	Dose level 1: 1 × 10^5^ cells/kg Dose level 2: 1 × 10^6^ cells/kg Dose level 3: 1 × 10^7^ cells/kg	Cyclophosphamide and fludarabine	None	No GvHD and ICANS were observed. one case developed CRS (grade I)	Overall: the day 30 and day 100 ORR (including PR and CR) were 48.6% for both. The day 30 and day 100 CR rates were 27% and 29.7%, respectively. The 1-year CR rate was 37.8%. 1-year CR rates for patients with FL and MZL were 83 (5/6) and for DLBCL was 29% (5/17)	Marin et al. [207]

*IL-15RF* IL-15 receptor fusion, *NHL* Non-Hodgkin lymphoma, *CLL* Chronic lymphocytic leukemia, *CLL-RT* Chronic lymphocytic leukemia-richter transformation, *DLBCL* Diffuse large B-cell lymphoma, *FL* Follicular Lymphoma, *MZL* Marginal zone lymphomas, *MCL* Mantle cell lymphoma, *LPL* Lymphoplasmacytic lymphoma, *PB* Peripheral blood, *UCB* Umbilical cord blood, *iPSC* Induced pluripotent stem cell, *ALL* Acute lymphoblastic leukemia, *CRS* Cytokine release syndrome, *ICANS* Immune associated neurological symptom, *GVHD* Graft-versus-host disease, *CR* Complete response, *PR* Partial response, *ORR* Overall response rate, *OS* Overall survival, *PD* Progression disease, *β2 M* β2 microglobulin, *CIITA* Class II transactivator, *EGFR* Epidermal growth factor receptor

Goodridge et al. created a CAR-NK product called FT596. This product was derived from iPSCs. The iPSCs were modified to consistently produce anti-CD19 CAR, a high affinity and non-cleavable CD16 Fc receptor, and a combination of a membrane-bound IL-15 and an IL-15Rα fusion protein. In a Raji xenograft mouse model, the combination of FT596 with rituximab resulted in a substantial increase in the elimination of Raji tumor cells. In addition, when a mouse model that had been engrafted with human CD34 cells, FT596 showed enhanced longevity and safety compared to primary CAR19 T cells [211]. This platform has been translated into a multicenter, phase I clinical trial as monotherapy or in combination with rituximab to treat patients with R/R B-cell lymphoma [210]. A total of 20 patients underwent two treatment regimens, including 10 in regimen A (FT596 alone) and 10 in regimen B (FT596 cells combined with rituximab). Among the 17 evaluable patients, clinical response was observed in 9 patients (5 from regimen A and 4 from regimen B), 7 of whom achieved CR. Notably, no dose-limiting toxicity, ICANS, or GVHD of any grade was observed. Interestingly, 2/4 of patients treated with CAR-T-cell therapy at doses ≥ 9 × 10^7^ cells/kg achieved CR [210]. An extended follow-up period will provide insight into the durability and efficacy of this platform. More recently, similar peripheral blood-derived anti-CD19 CAR-NK cells (named NKX019, a cryopreserved product utilizing OX40/CD3-ζ signaling domains and expressing a membrane-bound form of IL-15 for activation) were investigated in a phase I trial as a monotherapy for 19 patients with R/R B-cell malignancies. Patients received a daily lymphodepletion regimen of fludarabine and cyclophosphamide for 3 days. Next, they received three infusions of NKX019 at 3 dose levels, with doses ranging from 300 million to 1.5 billion cells per infusion. During the follow-up period, no dose-limiting toxicity, neurotoxicity, CRS or GVHD was reported. Among the 14 patients with NHL, 8 achieved CR; however, 3 patients with indolent lymphoma subsequently experienced relapse after a remission period of greater than 6 months [208, 212].

## What’s Next? CIML NK cells

NK cells following exposure to happens, viral infection or a combination of cytokines achieve memory properties. NK cells preactivated with IL-12/15/18 have been described as cytokine-induced memory-like (CIML) NK cells [213]. CIML NK cells present distinctive characteristics, such as high proliferative capacity, sensitivity to low doses of IL-2, increased IFN-γ production, resistance to TGF-β, elevated glycolysis, and oxidative phosphorylation, which distinguishes them from conventional NK cells (cNK cells) [214–217]. In addition, the long-term life span and adaptive immune features of CIML NK cells have drawn attention to the use of these cells in cancer immunotherapy. Recent findings from preclinical and clinical trials have shown that CIML NK-based immunotherapy has produced promising results and also offers a safe approach to preventing GVHD, CRS, and neurotoxicity [218]. In the context of hematological malignancies, CIML NK cell-based immunotherapy has aided in the discovery of novel treatments for various cancers, particularly myeloid disorders. Similarly, adoptively transferred CIML NK cells trigger CR in 44% of R/R acute myeloid leukemia (AML) patients [219].

Another clinical trial by Shapiro et al. revealed that CIML NK cell infusion into an immune-compatible microenvironment in posttransplant relapsed AML, MDS, and MPN patients resulted in satisfactory expansion and persistence [220]. Similarly, CIML NK cells injected into pediatric/young adults with post-HCT-relapsed AML patients significantly expand and persist in a compatible milieu. Furthermore, this clinical trial established that AML patients were treated with donor lymphocyte infusions (DLIs), and CIML NK cells showed promising outcomes [221].

Unlike for myeloid disease, the therapeutic approach involving CIML NK cells in lymphoid malignancies has received less attention. One of these few studies was performed on a rat model of T-ALL, namely, Roser leukemia (RL). In this in vivo experiment, RL was treated with cNK cells, and NK cells were stimulated with IL12/15/18 (CIML NK cells). Based on these results, RL is resistant to cNK cells but not to CIML NK cells. Therefore, CIML NK cells could be introduced as a possibility for immunotherapeutic clinical trials in T-ALL patients [222].

The role of CIML NK cells in lymphoma was studied by Ni et al. in mice injected with RMA-S lymphoma cells. Tumor-bearing mice were treated with IL-12/15/18–preactivated NK cells and IL-15–pretreated NK cells. The results highlighted that compared with IL-15–pretreated NK cells, IL-12/15/18–preactivated NK cells display greater frequency, persistence, proliferation, and functional killing activity at the tumor site [223]. In another study, Gang et al. further investigated CIML NK cell incorporation in lymphoma. They preactivated NK cells with IL-12/15/18 and then developed them to express the anti-CD19 CAR structure. The 19-CAR-CIML NK cells exhibited improved in vitro cytotoxicity against Raji cells and CD19^+^ primary lymphoma cells, as illustrated by elevated IFN-γ production and degranulation capacity. In addition, 19-CAR-CIML NK cells exhibited satisfactory durability, expansion, and effector function in a human lymphoma xenograft mouse model [224].

Finally, the challenges in NK cell-based immunotherapy in NHLs, the highly appreciated features of CIML NK cells, and promising results from current preclinical studies have prompted us to develop new therapeutic options based on CIML NK cells. Hopefully, we will witness a fundamental revolution in the management of patients with NHL.

## NK cell expansion

NK cells offer significant potential for immunotherapy in NHL treatment, but there are still obstacles to overcome to harness their full therapeutic benefits. There are still efforts to obtain a considerable number of NK cells for therapeutic purposes and to ensure that the obtained NK cells are fully functional and capable of effectively targeting and killing abnormal cells. This requires careful selection and expansion of NK cells, which can be technically challenging in the laboratory [225, 226]. Most PB-derived NK cell expansion protocols can be categorized into feeder-cell or feeder-free systems [227].

### Feeder cells

The production of a significant amount of NK cells from a small initial quantity relies on feeder cells. These feeder cells, whether naturally or through additional modifications, present ligands for NK cell receptors. When combined with cytokines, this interaction drives a substantial expansion of NK cells outside the body, enabling the generation of a large number of NK cells for therapeutic purposes [228]. Various types of cells, such as EBV-transformed lymphoblastoids and genetically engineered HEK293 or K562 cell lines, are utilized as feeder cells. Among these, genetically modified K562 cells are the most commonly employed [227]. For example, when a mixed lymphocyte population is infected with Epstein‒Barr virus (EBV) in vitro, it results in an immortalized cell line that exhibits characteristics similar to those of proliferating B cells. With the expression of different ligands (4-1BBL), CD155, CD48, and CD58) that have specific receptors (4-1BB, DNAM-1, 2B4, and CD2, respectively) on activated NK cells, EBV-bearing lymphoblastoid cell lines (LCLs) play essential roles in NK cell expansion and stimulation [228]. Using this method, an average of 1,000–2,000-fold expansion of NK cells was reported to be observed over a period of 14 days [229]. The addition of IL-21 and IL-2 reportedly improved the expansion efficacy [227, 230, 231]. In another method, irradiated feeder cells were employed to amplify NK cells in laboratory settings. The K562 leukemia cell line has been altered to display particular ligands linked to antigen-presenting cells (CD64, CD86, and truncated CD19, CD137L, 4-1BB ligand, and membrane-bound IL-21). The irradiated K562-mbIL21-41BBL cells seemed to be very effective at rapidly increasing the number of NK cells in RPMI media (containing 10% FBS). These modified cells expanded NK cells 47,967-fold in 21 days [232]. Nevertheless, using feeder cells can pose challenges due to licensing intricacies, difficulties in sourcing, and the requirement for their elimination from the culture. Challenges such as incomplete irradiation of feeder cells (which might lead to teratoma) and separating and thoroughly eliminating cancer cells from the culture environment to avoid injection into patients are additional difficulties [233].

### Feeder-free expansion methods

Expanding NK cells without the need for feeder cells has benefits compared to traditional methods, especially in terms of lower contamination risks and improved regulatory compliance. Additionally, other benefits, such as reducing costs through a more straightforward process and even decreasing cytotoxicity, have been reported [227]. Feeder-free NK cell expansion systems rely on cytokines and stimulating supplements or antibodies. Ex vivo cultured NK cells treated with IL-15 and nicotinamide exhibited stable CD62L expression, which was linked to increased FOXO1 levels. Nicotinamide enhanced NK cell metabolism, cytotoxicity, and cytokine production, leading to improved outcomes in adoptive transfer experiments. Recently, Cichocki et al. performed a phase 1 clinical trial in patients with relapsed or refractory NHL using rituximab in association with NK cells expanded with IL-15 and nicotinamide. The final result showed a 74% response rate in 19 patients [156]. Gluk et al. conducted two phase I studies to assess the combination therapy of rituximab and IL-2 (4.5–14 million international units) in relapsed or refractory B-cell NHL to boost ADCC through NK cell activation. The results showed that adding IL-2 to rituximab treatment is safe and effective, particularly with thrice-weekly IL-2 dosing, leading to increased NK cell counts and associated with treatment response [234]. In conclusion, obtaining a sufficient number of functional NK cells for therapeutic purposes remains a challenge. Despite the advancements made in feeder-free NK cell expansion, further investigations are needed to optimize this process and ensure its utility in clinical applications.

## Conclusion

In this comprehensive review, we first provided an overview of NK cells, including their function, characteristics, development, and maturation. We then delved into the complex tumor microenvironment and the interplay between various presented cells that can either support or hinder the antitumor activity of NK cells in NHL.

Building on these findings, we explored various strategies to enhance the therapeutic potential of NK cells because based on the findings reported in the literature, the function and number of NK cells are defective in NHL patients. Therefore, it would be beneficial to bolster the innate immune response by injecting and activating NK cells. Also, combinations of NK cells with multiplex immunotherapy strategies such as mAbs, BiKEs, and CARs could be effective and have been investigated in numerous clinical trials. The mAbs and BiKEs augmented NK cell-killing activity mediated by ADCC. However, BiKEs simultaneously bind to the tumor antigen and the NK cell surface Fc receptor, potentially creating a bridge between NK cells and tumor cells and allowing them to act more effectively than mAbs. The use of NK cells engineered with a CAR structure is another type of NK cell-based immunotherapy for NHLs. CAR-NK cells, when equipped with cytokine receptors or cytokine genes, have demonstrated enhanced proliferation and prolonged survival in the patient's bloodstream. They can target TAAs with particular specificity and result in improved treatment responses. CIML NK cells with adaptive immune characteristics and long lifespans are also appropriate for this application, but they have not been well assessed in NHLs.

By reviewing the available clinical trial data, we concluded that NK cell-based approaches are generally well-tolerated, with no major safety concerns observed specifically GVHD. Overall, the available clinical trial data provide an encouraging foundation for the continued investigation and development of NK cell-based immunotherapies for the management of NHLs. The safety profile demonstrated in these studies, coupled with the potential for improved clinical outcomes, warrants further exploration of NK cell-based approaches, either as standalone therapies or in combination with other modalities, to improve the treatment landscape for patients with this complex hematological malignancy.

## Data Availability

Not applicable.

## Abbreviations

HL: Hodgkin lymphoma
NHL: Non-Hodgkin lymphoma
mAb: Monoclonal antibody
SCT: Stem cell transplantation
CAR: Chimeric antigen receptor
R/R: Relapsed/refractory
CRS: Cytokine release syndrome
ICANS: Immune effector cell-associated neurotoxicity syndrome
GVHD: Graft-versus-host disease
NK cell: Natural killer cell
TME: Tumor microenvironment
DLBCL: Diffuse large B-cell lymphoma
MCL: Mantle cell lymphoma
BL: Burkitt lymphoma
FL: Follicular lymphoma
MZL: Marginal zone lymphoma
CLL/SLL: Chronic lymphocytic leukemia/small-cell lymphocytic lymphoma
HSCT: Hematopoietic stem cell transplantation
CTL: Cytotoxic T-lymphocyte
BiTE: Bispecific T cell engager
ILC: Innate lymphoid cell
HSC: Hematopoietic stem cell
MHC: Major histocompatibility complex
LGL: Large granular lymphocyte
TCR: T cell receptor
CLP: Common lymphoid progenitor
NKP: NK precursor
rIL: Recombinant interleukin
NKG2: Natural killer group 2
CCR: Chemokine (C–C motif) receptor
KIR: Killer-cell immunoglobulin-like receptor
DAP: DNAX-activation protein
NCR: Natural cytotoxicity receptor
ULBP: UL16-binding protein
MICA/B: MHC class I chain-related protein A and B
IDO: Indoleamine-pyrrole 2,3-dioxygenase
PGE2: Prostaglandin E2
DNAM-1: DNAX accessory molecule-1
GPI: Glycosylphosphatidylinositol
FcγRIII: IgG Fc region receptor III
ADCC: Antibody-dependent cell-mediated cytotoxicity
PD-1: Program-cell death receptor 1
PD-L1/2: PD-1/2 Ligand
Siglecs: Sialic acid recognizing immunoglobulin-like lectins
LAIR-1: Leukocyte-associated immunoglobulin-like receptor-1
TRAIL: TNF-related apoptosis-inducing ligand
FasL: Fas ligand
TRADD: TNF receptor-associated death domain
TH: Helper T cell
Treg: Regulatory T cell
DC: Dendritic cell
TAM: Tumor-associated macrophage
TAN: Tumor-associated neutrophil
MDSC: Myeloid-derived suppressor cell
TFH: Follicular helper T cell
MSC: Mesenchymal stromal cell
CAF: Cancer-associated fibroblast
ECM: Extracellular matrix
IFN: Interferon
TNF: Tumor necrosis factor
CTLA-4: Cytotoxic T lymphocyte-associated antigen 4
LAG-3: Lymphocyte activation gene 3
TGF: Tumor growth factor
CXCR: Chemokine (C-X-C motif) receptor
TFR: Follicular regulatory T cell
FoxP3: Forkhead box P3
TIM-3: T-cell immunoglobulin and mucin domain-containing 3
FDC: Follicular dendritic cell
GC: Germinal center
Ag-Ab complex: Antigen-Antibody complex
GM-CSF: Granulocyte-macrophage colony-stimulating factor
CXCL: Chemokine (C-X-C motif) ligand
APRIL: A proliferation-inducing ligand
ICAM-1: Intercellular adhesion molecule 1
VEGF: Vascular endothelial growth factor
M-MDSC: Monocytic MDSC
PMN-MDSC: Granulocytic MDSC
Arg1: Arginase1
iNOS: Inducible nitric oxide synthase
ROS: Reactive oxygen species
A-NKC: Absolute NK cell count
EFS: Event-free survival
PFS: Progression-free survival
OS: Overall survival
PCNSL: Primary central nervous system lymphoma
TIGIT: T-cell immunoreceptors with Ig and ITIM domain
HGBL: High‐grade B‐cell lymphoma
ILT2: Inhibitory receptors immunoglobulin-like transcript 2
CNS-DLBCL: DLBCL of the central nervous system
DD: Death domain
MALTL: Mucosa-associated lymphoid tissue lymphoma
CBCL: Cutaneous B-cell lymphoma
ALCL: Anaplastic large cell lymphoma
LFA-3: Lymphocyte-function antigen 3
HLA: Human leukocyte antigen
ABC-DLBCL: Activated B-cell-DLBCL
GC-DLBCL: Germinal center-DLBCL
GVT: Graft-versus-tumor
SD: Stable disease
FDA: Food and drug administration
CR: Complete response
PR: Partial response
ORR: Overall response rate
PB: Peripheral blood
CB: Cord blood
iPSC: Induced pluripotent stem cell
RFS: Relapse free survival
haNk: High affinity NK cell
MM: Multiple myeloma
iNK: NK cells derived from iPSCs
HSPC: Hematopoietic stem and progenitor cell
BiKE: Bispecific killer engager
scFv: Single-chain antibody variable fragment
TAA: Tumor-associated antigen
SLAM: Signaling lymphocyte activation molecule
PBMC: Peripheral blood mononuclear cell
cNK cell: Conventional NK cell
CIML NK cell: Cytokine-induced memory-like NK cells
AML: Acute myeloid leukemia
DLI: Donor lymphocyte infusion
RL: Roser leukemia
ALL: Acute lymphocytic leukemia
LCL: Lymphoblastoid cell line
